# MiR-290 Family Maintains Pluripotency and Self-Renewal by Regulating MAPK Signaling Pathway in Intermediate Pluripotent Stem Cells

**DOI:** 10.3390/ijms25052681

**Published:** 2024-02-26

**Authors:** Yueshi Liu, Xiangnan Li, Xiaozhuang Ma, Qiankun Du, Jiemin Wang, Haiquan Yu

**Affiliations:** State Key Laboratory of Reproductive Regulation and Breeding of Grassland Livestock (RRBGL), Inner Mongolia University, Hohhot 010070, China; liuyueshi@yeah.net (Y.L.); xiangnan_li@yeah.net (X.L.); xzma1998@163.com (X.M.); duqi416@163.com (Q.D.); jiemin_w@163.com (J.W.)

**Keywords:** pluripotency, naïve stem cells, primed stem cells, intermediate state, miR-290 family, p38 MAPK signaling pathway

## Abstract

Mouse embryonic stem cells (ESCs) and epiblast stem cells (EpiSCs) are derived from pre- and post-implantation embryos, representing the initial “naïve” and final “primed” states of pluripotency, respectively. In this study, novel reprogrammed pluripotent stem cells (rPSCs) were induced from mouse EpiSCs using a chemically defined medium containing mouse LIF, BMP4, CHIR99021, XAV939, and SB203580. The rPSCs exhibited domed clones and expressed key pluripotency genes, with both X chromosomes active in female cells. Furthermore, rPSCs differentiated into cells of all three germ layers in vivo through teratoma formation. Regarding epigenetic modifications, the DNA methylation of *Oct4*, *Sox2*, and *Nanog* promoter regions and the mRNA levels of *Dnmt3a*, *Dnmt3b*, and *Dnmt1* were reduced in rPSCs compared with EpiSCs. However, the miR-290 family was significantly upregulated in rPSCs. After removing SB203580, an inhibitor of the p38 MAPK pathway, the cell colonies changed from domed to flat, with a significant decrease in the expression of pluripotency genes and the miR-290 family. Conversely, overexpression of *pri-miR-290* reversed these changes. In addition, *Map2k6* was identified as a direct target gene of *miR-291b-3p*, indicating that the miR-290 family maintains pluripotency and self-renewal in rPSCs by regulating the MAPK signaling pathway.

## 1. Introduction

Pluripotent stem cells (PSCs) possess two remarkable properties: indefinite self-renewal capacity and pluripotency, which enable them to give rise to all tissues in the adult body. Pluripotency is a dynamic process that evolves during different stages of pre- and post-implantation development [1]. Consequently, the derivation and characterization of distinct pluripotent states in research on PSCs have become exciting areas of investigation [2]. Two types of pluripotent cells can be captured from a mouse embryo at different stages: mouse embryonic stem cells (ESCs) from the inner cell mass (ICM) of a blastocyst at E3.5, representing the naïve state of pluripotency, and epiblast stem cells (EpiSCs) from the late epiblast layer of a post-implantation embryo at E5.5–E7.5, representing the primed state of pluripotency [3,4]. Despite both referring to pluripotency, they differ markedly in morphology, gene expression profile, conditions for maintenance, epigenetic state, chimeric competence, and germline contribution [5]. PSCs provide invaluable in vitro cell models for understanding early mammalian development and hold great potential for regenerative medicine.

The maintenance and preservation of naive or primed PSCs can be achieved by modulating growth conditions in vitro. Naïve pluripotency in ESCs culture is supported by LIF and either serum or inhibitors of the Erk (PD0325901) and Gsk3 (CHIR99021) pathways (known as 2i) [6]. Using 2i/LIF captures naïve pluripotency, sometimes called the pluripotent ground state [7]. The primed pluripotency of EpiSCs is maintained using Activin A and fibroblast growth factor 2 (FGF2) [4,8]. Both types of cells are pluripotent, as they can differentiate into derivatives of all three germ layers in vivo by producing teratoma. However, only ESCs can produce chimeras when injected into a blastocyst [9]. Furthermore, a significant epigenetic difference between naïve and primed states of pluripotency is that female ESCs have two active X chromosomes, while female EpiSCs already present one inactive X chromosome [10].

Recently, a third phase called formative pluripotency has been proposed to exist as part of a developmental continuum between the naïve and primed phases [11,12,13]. Formative stem cells (FSCs) correspond to a post-implantation embryo at E5.5–E5.75. Nodal/Activin and FGF signals have been identified as candidate factors that support formative pluripotency [11,12]. Key transcription factors for formative pluripotency include *Otx2*, *Oct6*, and *Sox3*. Interestingly, *Otx2* is required for maintaining a stable formative state but is dispensable in both ESCs and EpiSCs [14]. Moreover, germ cell competency serves as a central feature to distinguish FSCs from primed EpiSCs, as FSCs directly respond to germ cell induction, unlike ESCs or EpiSCs [15,16].

The regulation of pluripotency involves an interplay of transcription factors, signaling pathways, micro-RNAs (miRNAs), and chromatin regulators. *Oct4*, *Sox2*, and *Nanog* are identified as core factors in the transcription factor network critical for maintaining the ESCs state [17,18,19]. LIF/Stat3, BMP4, Wnt/β-catenin, and FGF/Erk signaling pathways are involved in mouse pluripotency maintenance. Increasing evidence suggests that epigenetics has emerged as a crucial player in pluripotency maintenance, for instance, the knockout of DNA methyltransferase (Dnmt) in mouse ESCs causes DNA hypo-methylation, which can impact differentiation and lineage determination [20,21]. In addition, miRNAs are key regulators of self-renewal and differentiation in stem cells, holding as much importance as transcription factors in controlling gene expression [22].

Therefore, understanding the mechanisms underlying the different pluripotent states of PSCs, as well as studying how this unique property is retained, is essential not only for elucidating mammalian embryogenesis and cellular commitment but also for establishing therapies for regenerative medicine, disease modeling, and drug discovery. Herein, EpiSCs were successfully converted into a new type of reprogrammed PSCs (rPSCs) using a chemically defined medium consisting of LIF combined with BMP4, CHIR99021, XAV939, and SB203580. LIF and CHIR99021 improve cell culture robustness and support intermediate pluripotent cells for self-renewal [23,24]. Ying and Yu et al. demonstrate that BMP4 inhibits differentiation genes, sustains self-renewal in mouse ESCs in collaboration with STAT3, and is also essential for primed to naïve transition [25,26]. Furthermore, MAPK inhibitors support naïve pluripotency in mouse ESCs [27], while XAV939 suppresses Yap1 activity, potentially modulating multiple targets relevant to trophectoderm/inner cell mass segregation [28]. In this study, we assessed the pluripotency and differentiation potential of rPSCs while further investigating the potential mechanism of the miR-290 family in maintaining rPSCs pluripotency.

## 2. Results

### 2.1. Conversion of EpiSCs to rPSCs

**Figure 1:**
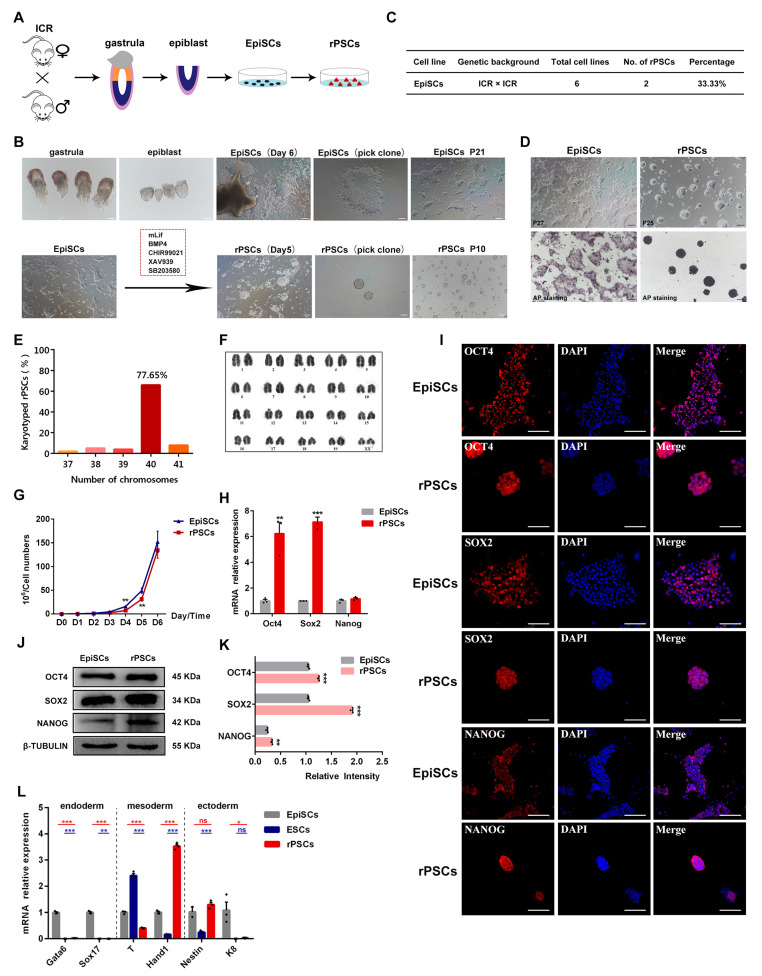
Derivation and characterization of rPSCs. (**A**) Schematic of the derivation of rPSCs. (**B**) A bright field image shows the derivation process of rPSCs. Scale bars: 100 µm. (**C**) Efficiency of rESCs conversed with EpiSCs in rPSCs medium. (**D**) Alkaline phosphatase (AP) staining on rPSCs and EpiSCs. Scale bars: 100 µm. (**E**) Distribution of chromosome numbers in rPSCs. (**F**) Karyotype of rPSCs. XX is two X chromosomes, representing the female cell line. (**G**) Cell growth curves of rPSCs and EpiSCs. (**H**) RT-qPCR analysis of pluripotency gene expression in rPSCs, and EpiSCs were used as a control. (**I**) Immunofluorescence staining of OCT4, SOX2, and NANOG in rPSCs and EpiSCs. Scale bars: 50 µm. (**J**) Western blot analysis for OCT4, SOX2, and NANOG in rPSCs and EpiSCs. (**K**) Quantification of OCT4, SOX2, and NANOG protein intensity analysis in rPSCs and EpiSCs. (**L**) RT-qPCR analysis of endoderm-, mesoderm-, and ectoderm-associated gene expression in rPSCs, EpiSCs, and ESCs. The above experiments included three replications. Error bars are SEM. Significance was tested with two-tailed Student’s *t*-tests, with * *p* < 0.05, ** *p* < 0.01, *** *p* < 0.001, and ns at *p* > 0.05.

Mouse EpiSCs, derived from early post-implantation embryos, exhibit distinct culture properties, gene expression, pluripotency, and epigenetic profiles compared with ESCs [4,8]. In this study, EpiSCs were derived from the epiblast tissue of female mice at E6.5 and cultured in a chemically defined medium containing Activin A and bFGF, without mouse embryonic fibroblasts (MEFs) or serum. Subsequently, EpiSCs were transferred to rPSCs medium consisting of basic N2B27 medium supplemented with mouse LIF, BMP4, CHIR99021, XAV939, and SB203580 (Figure 1A,B). After 5–7 days of culture, the domed colonies resembling ESCs morphology gradually formed (Figure 1B and Figure S1A). Domed colonies were selected with a glass needle for mechanical propagation and successfully developed into self-renewal stem cell lines for more than 30 passages in rPSCs medium. And the derivation efficiency of rPSCs was 33.3% (Figure 1C). The induced stem cells from rPSCs medium were named rPSCs, and they exhibited positive alkaline phosphatase (AP) staining, similar to ESCs, indicating their self-renewal capacity (Figure 1D and Figure S1A). The karyotype analysis showed that rPSCs maintained a normal karyotype (77.65%) (Figure 1E,F). Growth curve analysis revealed that rPSCs exhibited a proliferation rate similar to EpiSCs but faster than ESCs (Figure 1G and Figure S1B).

To characterize rPSCs, we examined the expression levels of primed pluripotency and naïve pluripotency markers. RT-qPCR analysis of rPSCs revealed high expression levels of pluripotency genes, such as *Oct4* and *Sox2*, compared with EpiSCs or ESCs (Figure 1H and Figure S1C). Interestingly, rPSCs exhibited intermediate expression levels of markers related to naïve pluripotency, such as *Klf4* and *Dppa*4, between those of EpiSCs and ESCs, while the primed pluripotency gene *Fgf5* was barely expressed in rPSCs compared with EpiSCs (Figure S1D). Immunofluorescence assays showed that rPSCs can express OCT4, SOX2, and NANOG (Figure 1I and Figure S1E). And Western blot analysis demonstrated significantly higher protein expression levels of OCT4, SOX2, and NANOG in rPSCs compared with EpiSCs, resembling the expression levels observed in ESCs (Figure 1J,K and Figure S1F,G). In addition, RT-qPCR analysis revealed significantly decreased expression of endoderm markers (*Gata6* and *Sox17*) and ectoderm marker *K8* in rPSCs, while the expression of the mesoderm marker *Hand1* was upregulated in rPSCs (Figure 1L).

Taken together, our results demonstrate that a chemically defined medium supplemented with LIF, BMP4, CHIR99021, XAV939, and SB203580 can induce the conversion of EpiSCs to rPSCs. rPSCs represent novel and distinct self-renewal type of PSCs that stably express pluripotency markers and display molecular properties similar to ESCs.

### 2.2. Developmental and Differentiated Potency of rPSCs

**Figure 2:**
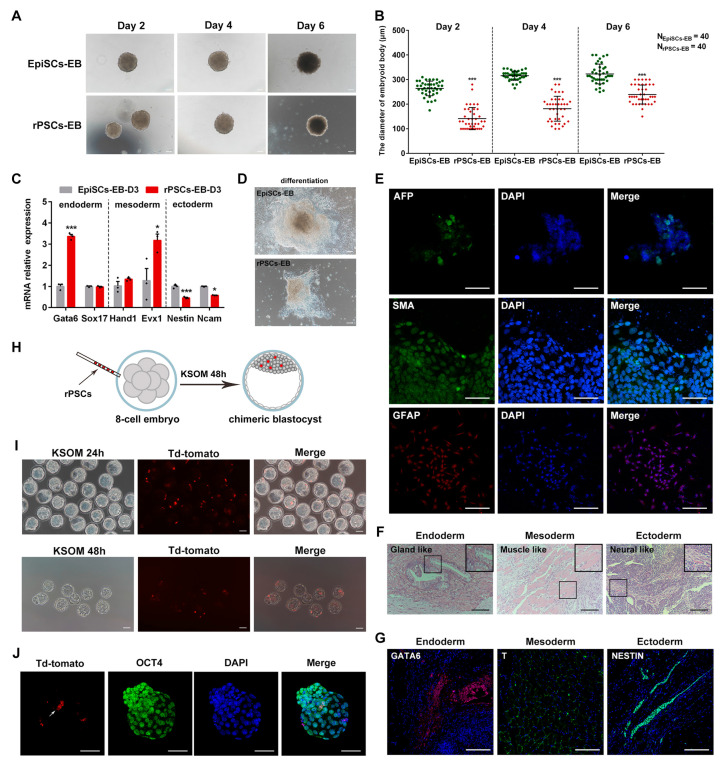
Differentiated and developmental potencies of rPSCs. (**A**) Morphology of embryoid body (EB) induction by rPSCs and EpiSCs on Day 2, Day 4, and Day 6. Scale bars: 100 µm. (**B**) Statistics of the number and diameter of EB formed by rPSCs and EpiSCs. N represents the number of EB spheres. (**C**) RT-qPCR analysis of endoderm-, mesoderm-, and ectoderm-associated gene expression in the EB of rPSCs and EpiSCs. (**D**) Adherence differentiation of EB formed by rPSCs and EpiSCs for 7 days. Scale bars: 100 µm. (**E**) Immunofluorescence staining of three germ layer markers in the EB of rPSCs and EpiSCs. AFP was used to visualize the endoderm, SMA was used to visualize the mesoderm, and GFAP was used to visualize the ectoderm. Scale bars: 50 µm. (**F**) Mature teratoma from rPSCs. Left: endoderm, gland-like cells. Middle: mesoderm, muscle-like cells. Right: ectoderm, neural-like cells. The sections were stained with H&E. Scale bars: 100 µm. (**G**) Immunofluorescence staining of three germ layer markers in the teratoma test. GATA6 was used to visualize the endoderm, T was used to visualize the mesoderm, and NESTIN was used to visualize the ectoderm. Scale bars: 50 µm. (**H**) Schematic of the eight-cell embryo injection protocol. (**I**) E3.5 chimeras generated by injecting rPSCs into eight-cell embryos and cultured in KSOM for 24 h and 48 h. Scale bars: 100 µm. (**J**) Immunofluorescence staining of blastocysts at 24 h after injection of rPSCs. OCT4 was used as the ICM marker. Scale bars: 50 µm. The above experiments included three replications. Error bars are SEM. Significance was tested with two-tailed Student’s *t*-tests, with * *p* < 0.05 and *** *p* < 0.001.

To assess the differentiated potency of rPSCs in vitro, we compared the formation of embryoid body (EB) between rPSCs, EpiSCs, and ESCs. Suspension culture was used to allow for the formation of cell clusters during spontaneous differentiation, mimicking developmental processes in vivo [29]. Our results revealed that the EB derived from rPSCs exhibited a smaller size at day 2 compared with the spherical EB derived from EpiSCs or ESCs (Figure 2A and Figure S2A). To further verify this difference, we employed the pendant-drop method to form EB. The diameter of rPSCs-derived EB (rPSCs-EB) at day 6 was approximately 250 μm, while EpiSCs-derived EB (EpiSCs-EB) and ESCs-derived EB (ESCs-EB) had diameters of around 330 μm and 300 μm, respectively (Figure 2B and Figure S2B). We also examined the expression of three germ layer markers. RT-qPCR results showed that the expression of endoderm marker *Gata6* and mesoderm markers *Hand1* and *Evx1* in rPSCs-EB was higher than EpiSCs-EB but lower than ESCs-EB (Figure 2C and Figure S2C). In contrast, the ectoderm markers *Nestin* and *Ncam* were downregulated in rPSCs-EB compared with EpiSCs-EB (Figure 2C). Furthermore, we cultured the EB on a 24-well plate for 7 days, and immunofluorescence staining revealed that EB-differentiated cells can express the endoderm marker AFP, the mesoderm marker SMA, and the ectoderm marker GFAP (Figure 2D,E).

In addition, similar to ESCs and EpiSCs, rPSCs also generated teratoma containing derivatives of the three germ layers (Figure 2F,G and Figure S2D). To further investigate the pluripotency of rPSCs, we performed chimera tests in vivo. rPSCs were transfected with the H2B td-Tomato plasmid, and eight rPSCs were injected into 8-cell-stage mouse embryos. The embryos were then cultured in vitro for 48 h, and immunofluorescence staining showed that rPSCs can contribute to the ICM (Figure 2H–J). In contrast, EpiSCs exhibit inefficiency in contributing to chimeric tissues following blastocyst injection and display less active expression of genes related to naïve pluripotency [8]. These findings suggest that rPSCs have the potential to contribute to embryos. Taken together, our data demonstrate that the rPSCs exhibit greater developmental and differentiated potency compared with EpiSCs.

### 2.3. Molecular Features of rPSCs

**Figure 3:**
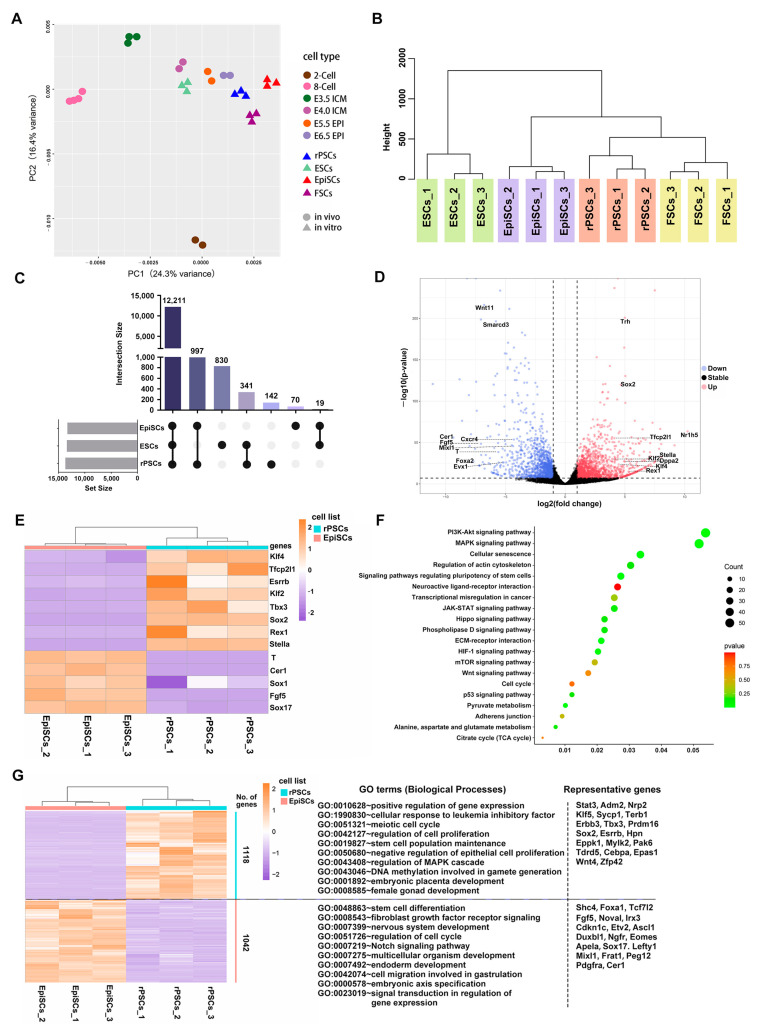
Analyses of molecular features of rPSCs. (**A**) Principal component analysis (PCA) of transcriptome data from pre-implantation embryos (2-Cell, 8-Cell, E3.5 ICM, and E4.0 ICM), post-implantation (E5.5 EPI and E6.5 EPI), ESCs, EpiSCs, FSCs, and rPSCs. (**B**) Hierarchical clustering of the transcriptome from three biological replicates (*n* = 3) of four pluripotent stem cell lines. (**C**) The UpSet plot shows specific genes in three pluripotent stem cell lines. (**D**) The volcano plot of differentially expressed genes for rPSCs versus EpiSCs. (**E**) Heatmap showing scaled expression of pluripotency transcription factors and lineage factors. (**F**) Kyoto Encyclopedia of Genes and Genomes (KEGG) pathway analysis of DEGs for rPSCs versus EpiSCs described in (**D**). (**G**) Heatmap showing differentially expressed genes (fold change of ≥2, adjusted *p* value < 0.001) in rPSCs (*n* = 3) compared with EpiSCs (*n* = 3). Significantly enriched gene ontology (GO) terms and representative genes in each cluster are listed on the right.

To better understand the molecular features of rPSCs, we performed RNA sequencing (RNA-seq) to analyze the transcriptomes of rPSCs. Principal component analysis (PCA) results revealed that the global gene expression patterns of rPSCs were close to formative stem cells (FSCs) and appeared to be at an intermediate state between naïve ESCs and formative FSCs but distinct from primed EpiSCs (Figure 3A). In addition, compared with pre- and post-implantation embryos in vivo [30,31,32], naïve ESCs clustered with pre-implantation E4.0 ICM, rPSCs, and FSCs clustered with post-implantation E6.5 EPI, while EpiSCs had progressed to more advanced stages (Figure 3A). Hierarchical cluster analysis also revealed that rPSCs and formative FSCs cluster together (Figure 3B), showing that rPSCs appeared to be in an intermediate state between naïve ESCs and formative FSCs but distinct from primed EpiSCs (Figure 3A,B). The UpSet plot identified 142 unique genes in rPSCs (Figure 3C). This distinct clustering pattern of rPSCs was further confirmed by constructing a correlation matrix of gene expression clustered using Pearson correlation coefficients (Figure S3A). A violin plot illustrated the expressed genes (Figure S3B), and differentially expressed genes between rPSCs and EpiSCs were analyzed using the DEseq2 R package (*p*-value < 0.01 and fold change of ≥2) to examine molecular differences.

We identified 4514 differentially expressed genes between rPSCs and EpiSCs, with 2573 genes significantly upregulated, including naïve pluripotency markers (Klf2, Klf4, Rex1, Sox2, and Dppa2), and 1941 genes significantly downregulated, mainly representing lineage factors (Fgf5, T, Evx1, Foxa2, and Cer1) (Figure 3D). This suggests distinct transcriptional patterns between rPSCs and EpiSCs. The expression of pluripotency transcription factors was higher in rPSCs compared with EpiSCs, while primed pluripotency markers showed significant downregulation in rPSCs (Figure 3E and Figure S3C). Kyoto Encyclopedia of Genes and Genomes (KEGG) pathway enrichment analysis revealed the top 20 enriched pathways, including “signaling pathways regulating pluripotency of stem cells”, “PI3K-Akt signaling pathway”, “MAPK signaling pathway”, and “cell cycle” (Figure 3F). Applying a cutoff of adjusted *p*-value < 0.001 and fold change of ≥2, we identified 1118 upregulated genes and 1042 downregulated genes in rPSCs compared with EpiSCs (Figure 3G). The upregulated genes in rPSCs were associated with “regulation of cell proliferation”, “stem cell population maintenance”, “DNA methylation involved in gamete generation”, and “embryonic placenta development” with representative genes such as Tbx3, Prdm16, Pak6, Cebpa, and Epas1, while the downregulated genes in rPSCs were primarily enriched in “stem cell differentiation”, “nervous system development”, and “endoderm development”, with representative genes including Foxa1, Tcf7l2, Sox17, Irx3, and Pdgfra (Figure 3G).

Collectively, rPSCs and EpiSCs exhibited distinct gene expression patterns. Most of the upregulated genes in rPSCs were predominantly developmental regulatory genes, cell proliferation regulatory genes, and genes associated with stem cell pluripotency signaling pathways. These data indicate that the molecular features of rPSCs represent an intermediate state between EpiSCs and ESCs.

### 2.4. Epigenetic Changes in rPSCs

**Figure 4:**
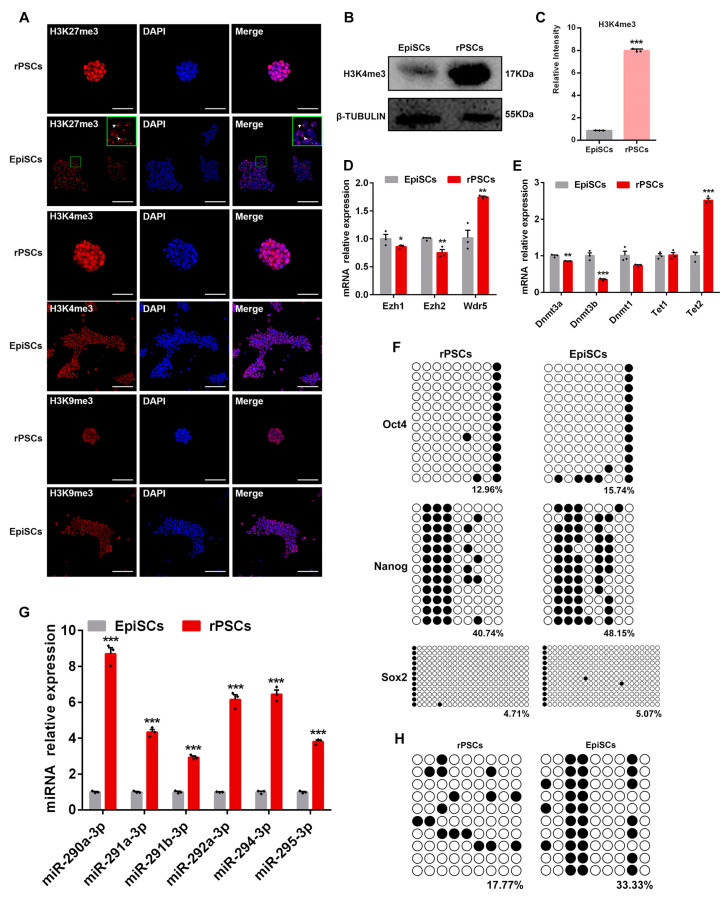
Epigenetic changes during the conversion of EpiSCs to rPSCs. (**A**) Immunofluorescence staining of H3K27me3, H3K4me3, and H3K9me3 in rPSCs and EpiSCs. White arrows point to the bright H3K27me3 foci in female EpiSCs. Scale bars: 50 µm. (**B**) Western blot analysis for H3K4me3 protein in rPSCs and EpiSCs. (**C**) Quantification of H3K4me3 protein intensity analysis in rPSCs and EpiSCs. (**D**) RT-qPCR analysis of *Ezh1*, *Ezh2*, and *Wdr5* expression in rPSCs and EpiSCs. (**E**) RT-qPCR analysis of DNA methylation-related gene expression (*Dnmt3a*, *Dnmt3b*, *Dnmt1*, *Tet1*, and *Tet2*) in rPSCs, and EpiSCs were used as a control. (**F**) Changes in DNA methylation of *Oct4*, *Sox2*, and *Nanog* promoter regions during the conversion of EpiSCs to rPSCs. (**G**) RT-qPCR analysis of mature miRNAs *miR-290-3p*, *miR-291a-3p*, *miR-291b-3p*, *miR-292a-3p*, *miR-294-3p*, and *miR-295-3p* in rPSCs, and EpiSCs were used as a control. (**H**) Changes in DNA methylation of the miR-290 super enhancer (SE) region in rPSCs and EpiSCs. The above experiments included three replications. Error bars are SEM. Significance was tested with two-tailed Student’s *t*-tests, with * *p* < 0.05, ** *p* < 0.01, and *** *p* < 0.001.

Next, we examined the epigenetic changes occurring during the reprogramming of EpiSCs to rPSCs. Notably, an important epigenetic change is the reactivation of the late-replicating, inactive X chromosome in the epiblast [33], which indicates a major epigenetic change like reprogramming [34]. E6.5 epiblast cells exhibit characteristic accumulation of histone H3 lysine 27 trimethylation (H3K27me3) epigenetic mark, which is diagnostic for the inactive X [35,36]. In addition, it has been reported that both X chromosomes are active in female naïve ESCs [10,37]; concurrent with this, our immunofluorescence showed bright H3K27me3 foci in female EpiSCs but not in female rPSCs, suggesting reactivation of the X-chromosome in rPSCs (Figure 4A). These observations showed continuing epigenetic reprogramming during the conversion of EpiSCs to rPSCs in a novel medium, where none of the cells had the characteristic H3K27me3 foci. The expression of proteins H3K4me3 and H3K9me3 was also detected by immunofluorescence, and we found that there was no significant difference in the H3K9me3 expression (Figure 4A). However, immunofluorescence and Western blot analysis revealed a significantly higher expression of H3K4me3 in rPSCs compared with EpiSCs (Figure 4A–C). Furthermore, we examined the expression of histone H3K27 methyltransferase Ezh1, Ezh2, and histone H3K4 methyltransferase Wdr5. RT-qPCR results showed that the expression of Ezh1 and Ezh2 was decreased, while that of Wdr5 was upregulated in rPSCs compared with EpiSCs (Figure 4D).

DNA methylation plays a critical role in embryonic development, stem cell differentiation, and cell fate conversion [38,39]. To investigate whether the reprogramming process of EpiSCs altered DNA methylation levels, we examined the mRNA expression levels of DNA methyltransferases (DNMTs) and the Tet family in EpiSCs and rPSCs. RT-qPCR analysis revealed a significant decrease in the expression of Dnmt3a, Dnmt3b, and Dnmt1 in rPSCs compared with EpiSCs, whereas Tet2 was found to be upregulated in rPSCs (Figure 4E). Previous studies have reported that Tet1 and Tet2 regulate 5hmC levels in mouse ESCs and are associated with the pluripotent state [40]. We further investigated the epigenetic changes in DNA methylation of the promoter regions of three key pluripotency genes, Oct4, Sox2, and Nanog. Bisulfite sequencing indicated that the Oct4 and Sox2 promoter regions were almost completely demethylated in rPSCs, EpiSCs, and ESCs. In contrast, the Nanog promoter region showed considerable methylation in EpiSCs compared with rPSCs and ESCs (48.15% vs. 40.74% vs. 39.8%) (Figure 4F and Figure S4A).

Several studies have demonstrated that the expression of the ESC-specific miRNAs is necessary for initiating stem cell differentiation and normal embryonic development [41]. ESC-specific miR-290–295 cluster accounts for 60% of the miRNA population in mouse ESCs [42,43] and is rapidly downregulated during differentiation [44,45]. The expression level of the miR-290 family varies among different types of stem cells. The expression level of the miR-290 family was significantly higher in mouse ESCs than that in trophoblast stem cells (TS) and extraembryonic endoderm cells (XEN), while it was barely expressed in MEFs [46]. Therefore, we examined the expression changes of the miR-290 family during the conversion of EpiSCs to rPSCs. RT-qPCR analysis revealed significant upregulation of miR-290 family members (miR-290a-3p, miR-291a-3p, miR-291b-3p, miR-292a-3p, miR-294-3p, and miR-295-3p) in rPSCs compared with EpiSCs (Figure 4G). Similarly, their expression levels were also higher than in ESCs (Figure S4B). The methylation of super enhancer (SE) regions has been shown to change during differentiation. Stelzer et al. reported that the miR-290 SE region is hypo-methylated in ESCs but becomes de novo methylated upon differentiation [47]. Bisulfite sequencing displayed that the methylation level at the miR-290 SE region in rPSCs is intermediate between EpiSCs and ESCs, consistent with previous studies (Figure 4H and Figure S4C).

These results suggest that rPSCs exhibit specific epigenetic features that are more similar to ESCs than to EpiSCs. We hypothesize that the enhanced pluripotency of rPSCs may be related not only to the regulation of transcription factors but also to the signaling pathways involved in the miR-290 family.

### 2.5. Removal of SB203580 Reduces the Pluripotency of rPSCs

**Figure 5:**
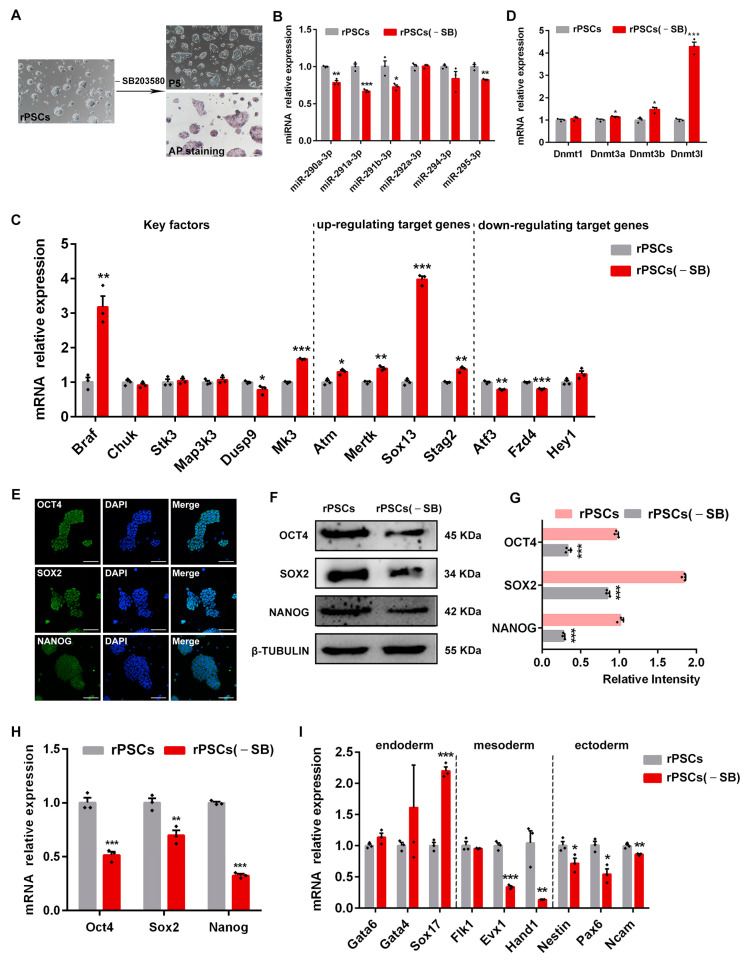
Removal of SB203580 reduced the pluripotency of rPSCs. (**A**) Morphological changes and AP staining after rPSCs removal of SB203580. Scale bars: 100 µm. (**B**) RT-qPCR analysis of mature miRNAs *miR-290-3p*, *miR-291a-3p*, *miR-291b-3p*, *miR-292a-3p*, *miR-294-3*p, and *miR-295-3p* expression in rPSCs and rPSCs(-SB). (**C**) RT-qPCR analysis of key factors and up-/down-regulating target gene expression of the p38 MAPK signaling pathway in rPSCs and rPSCs(-SB). (**D**) RT-qPCR analysis of DNA methylation-related gene expression in rPSCs and rPSCs(-SB). (**E**) Immunofluorescence staining of OCT4, SOX2, and NANOG in rPSCs(-SB). Scale bars: 50 µm. (**F**) Western blot analysis for OCT4, SOX2, and NANOG in rPSCs and rPSCs(-SB). (**G**) Quantification of OCT4, SOX2, and NANOG protein intensity analysis in rPSCs and rPSCs(-SB). (**H**) RT-qPCR analysis of pluripotency-associated gene expression in rPSCs and rPSCs(-SB). (**I**) RT-qPCR analysis of endoderm, mesoderm, and ectoderm-associated gene expression in rPSCs and rPSCs(-SB). The above experiments included three replications. Error bars are SEM. Significance was tested with two-tailed Student’s *t*-tests, with * *p* < 0.05, ** *p* < 0.01, and *** *p* < 0.001.

We further investigated the role of the MAPK signaling pathway in maintaining the pluripotency of rPSCs. SB203580, a p38 MAPK inhibitor, has been used in combination with various small-molecule compounds to promote the generation of mouse expended pluripotent stem cells (EPSCs) [48]. When rPSCs were cultured in the medium lacking SB203580, we observed a transition in cell morphology from domed to a smooth, flat shape similar to EpiSCs, accompanied by a reduction in AP positivity, which we refer to as rPSCs(-SB) (Figure 5A). Next, we examined whether the removal of SB203580 affected the expression of miR-290 family members. RT-qPCR analysis revealed significant downregulation of miR-290 family members, including *miR-290a-3p*, *miR-291a-3p*, *miR-291b-3p*, and *miR-295-3p* (Figure 5B). Notably, RNA-seq analysis of rPSCs showed that the KEGG pathway analysis was mainly concentrated in the MAPK signaling pathway (Figure 3F). Therefore, we investigated the effect of SB203580 removal on the MAPK signaling pathway by examining key factors and up-/down-regulating target genes of the p38 MAPK signaling pathway. RT-qPCR results showed that after removing SB203580, the expression of *Braf* and *Mk3* significantly increased, while *Dusp9* expression decreased. Furthermore, up-regulating target genes of the p38 MAPK signaling pathway (*Atm*, *Mertk*, *Sox13*, and *Stag2*) were upregulated, while down-regulating target genes (*Atf3*, *Fzd4*, and *Hey1*) were downregulated in rPSCs(-SB), indicating the activation of the p38 MAPK signaling pathway (Figure 5C).

Cells under different culture conditions exhibit different methylation patterns [49,50]. Therefore, we explored whether the removal of SB203580 altered DNA methylation levels. The expression of DNA methyltransferases *Dnmt3a*, *Dnmt3b*, and *Dnmt3l* was significantly upregulated after SB203580 removal (Figure 5D). To address whether SB203580 removal modulated the expression of key pluripotency genes, we performed immunofluorescence staining, RT-qPCR, and Western blot analysis. Immunofluorescence assays confirmed the expression of pluripotency markers OCT4, SOX2, and NANOG in rPSCs(-SB) (Figure 5E). Furthermore, Western blot analysis showed a significant decrease in protein expression of OCT4, SOX2, and NANOG in rPSCs(-SB) (Figure 5F,G). These findings were confirmed using RT-qPCR results (Figure 5H). Interestingly, rPSCs(-SB) exhibited higher expression of endoderm markers *Gata4* and *Sox17*, suggesting a tendency toward endodermal differentiation after the removal of SB203580 (Figure 5I). In contrast, the mesoderm markers *Evx1* and *Hand1* were downregulated. Similarly, the ectoderm markers *Pax6*, *Ncam*, and *Nestin* were also downregulated (Figure 5I).

Taken together, our results suggest that the removal of SB203580 leads to the inhibition of the p38 MAPK signaling pathway, resulting in a reduction in cell pluripotency and the induction of endoderm differentiation. Additionally, the expression of miR-290 family members is also decreased.

### 2.6. Map2k6 Is a Direct Target of miR-291b-3p

**Figure 6:**
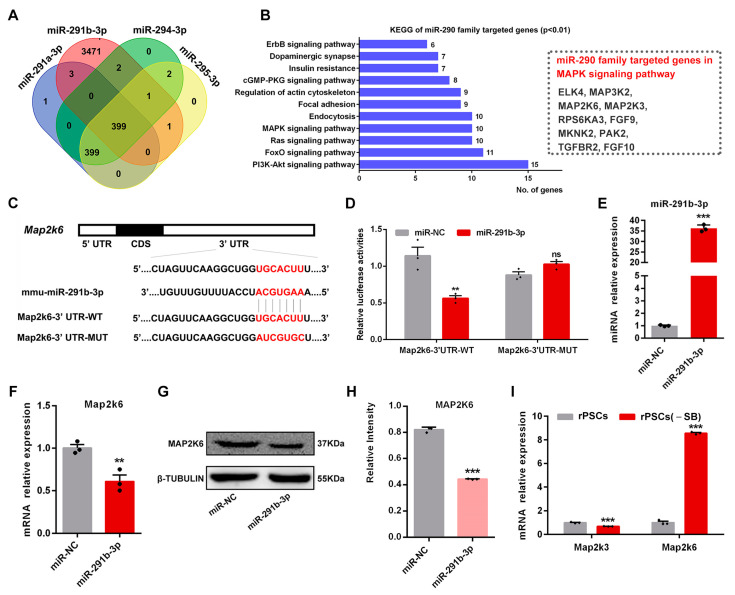
*Map2k6* is a direct target of *miR-291b-3p*. (**A**) Venn diagram of common targets of *miR-291a-3p*, *miR-291b-3p*, *miR-294-3p,* and *miR-295-3p*. (**B**) KEGG analysis showed that common targets were mainly enriched in the PI3K-AKT signaling pathway and the MAPK signaling pathway. (**C**) Target binding site of *miR-291b-3p* in the *Map2k6* mRNA 3′-UTR. CDS, coding sequence; WT, wild-type seed sequence; MUT, mutant seed sequence. (**D**) Relative luciferase activity in ESCs co-transfected with *miR-291b-3p* mimic and Map2k6-3′UTR-WT or Map2k6-3′UTR-MUT luciferase reporter vector. Each experiment included 1 × 10^6^ cells. (**E**,**F**) RT-qPCR analysis of the expression levels of *Map2k6* mRNA and *miR-291b-3p* in ESCs after *miR-291b-3p* mimic transfection. (**G**) Western blot analysis for MAP2K6 protein level in ESCs after *miR-291b-3p* mimic transfection. (**H**) Quantification of MAP2K6 protein intensity analysis in ESCs after *miR-291b-3p* mimic transfection. (**I**) RT-qPCR analysis of *Map2k3* and *Map2k6* expression in rPSCs and rPSCs(-SB). The above experiments included three replications. Error bars are SEM. Significance was tested with two-tailed Student’s *t*-tests, with ** *p* < 0.01, *** *p* < 0.001, and ns at *p* > 0.05.

Mature miRNAs typically recognize their mRNA targets through imperfect sequence complementarity and downregulate the target mRNA expression through a combined mechanism of translational inhibition and mRNA degradation [51]. To identify potential mRNA targets of miR-290 family members, including miR-291a-3p (707 genes), miR-291b-3p (3877 genes), miR-294-3p (708 genes), and miR-295-3p (707 genes), which all share the common seed sequence AAGUGC, we searched the miRBase database. A total of 399 overlapping target genes were identified (Figure 6A). KEGG pathway analysis revealed that these genes were primarily enriched in the “PI3K-AKT signaling pathway” and “MAPK signaling pathway” (Figure 6B). In addition, GO analysis was performed to explore the potential roles of these genes. In GO biological processes (GO-BP), the genes were enriched in terms such as “regulation of transcription, DNA-templated” and “transcription, DNA-templated” (Figure S5A). In GO cellular components (GO-CC), the genes were primarily enriched in the “nucleus” and “cytoplasm” (Figure S5B). In GO molecular functions (GO-MF), the genes were enriched in the term “protein binding” (Figure S5C).

Since SB203580 functions as an inhibitor of the p38 MAPK signaling pathway, we sought to explore the relationship between miR-290 and this pathway. KEGG pathway analysis revealed an enrichment of 10 genes in the MAPK signaling pathway; Map2k3, Map2k6, and Mnk2 were associated with the p38 MAPK signaling pathway (Figure 6B). Additionally, we focused on *miR-291b-3p* to identify potential mRNA targets of the miR-290 family using an online database available at https://www.targetscan.org (accessed on 5 December 2022), and we found that *Map2k3* and *Map2k6* were potential targets of mature *miR-291b-3p*. To confirm this hypothesis, we designed the wild-type 3′-UTR sequence for *Map2k6* cDNA (Map2k6-3′-UTR-WT) and a mutant 3′-UTR *Map2k6* cDNA sequence (Map2k6-3′-UTR-MUT) that would be unable to bind *miR-291b-3p*, as listed in Supplementary Table S2. These sequences were cloned into the luciferase reporter vector pmirGLO using *Xho* I/*Xba* I restriction enzymes (Figure 6C), and the recombinant plasmids were verified by sequencing (Figure S5D). Similar to *Map2k6*, Map2k3-3′-UTR-WT and Map2k3-3′-UTR-MUT sequences were also designed (Figure S5E) and cloned into pmirGLO vectors, followed by verification with sequencing (Figure S5D), as listed in Supplementary Table S2. The recombinant luciferase reporter vectors were co-transfected with *miR-291b-3p* mimic into ESCs, and subsequent measurement of luciferase activities revealed a significant decrease when the *miR-291b-3p* mimic was co-expressed with the Map2k6-3′-UTR-WT pmirGLO vector compared with the Map2k6-3′-UTR-MUT pmirGLO vector (Figure 6D). However, there were no differences in luciferase activities between the Map2k3-3′-UTR-WT and Map2k3-3′-UTR-MUT groups when co-expressed with *miR-291b-3p* mimic (Figure S5F). RT-qPCR assays were also performed to determine the regulatory relationship between *miR-291b-3p* and *Map2k6*. The *miR-291b-3p* mimic transfected into ESCs expressed higher levels of *miR-291b-3p* and lower levels of *Map2k6* compared with the control miR-NC group (Figure 6E,F); however, there was no distinct difference in *Map2k3* expression (Figure S5G). Western blot analysis revealed a significant decrease in the MAP2K6 protein level in ESCs after transfection with *miR-291b-3p* mimics (Figure 6G,H). We also examined the expressions of *Map2k3* and *Map2k6* before and after the SB203580 withdrawal. RT-qPCR results showed that *Map2k6* expression was significantly increased in rPSCs(-SB) compared with rPSCs, while the expression of *Map2k3* was slightly reduced (Figure 6I). These results demonstrated that Map2k6 was the direct downstream target gene of *miR-291b-3p*.

### 2.7. MiR-290 Family Can Replace MAPK Pathway Inhibitor SB203580 to Maintain the Pluripotency of rPSCs

MiRNAs can act as part of a pluripotency regulation network. Pluripotency-promoting miRNAs function to dampen differentiation signals during self-renewal by reinforcing the pluripotent state set by the pluripotency transcription factor and epigenetic regulation networks [52]. For instance, miR-302 suppresses epigenetic regulators *Aof2*, *Aof1*, *Mecp1-p66*, and *Mecp2* to trigger global demethylation [53]. This demethylation process is a prerequisite for establishing pluripotency.

**Figure 7:**
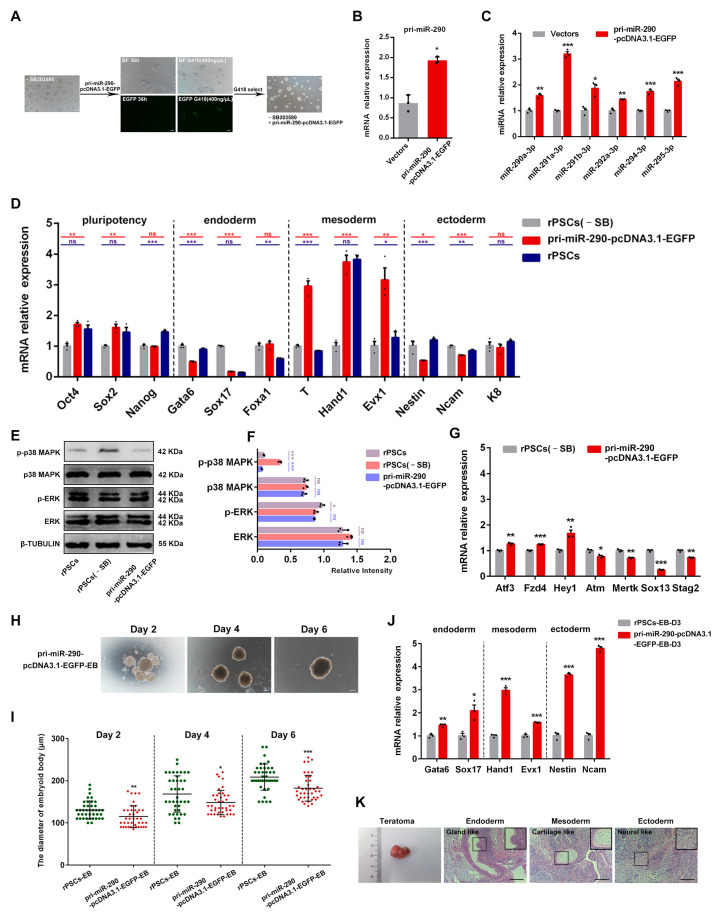
Overexpression of *pri-miR-290* can improve the pluripotency of rPSCs(-SB). (**A**) Morphological changes in rPSCs(-SB) after overexpression of *pri-miR-290*. Scale bars: 100 µm. (**B**) RT-qPCR was used to detect the overexpression efficiency of *pri-miR-290*. (**C**) RT-qPCR analysis of mature miRNAs *miR-290-3p*, *miR-291a-3p*, *miR-291b-3p*, *miR-292a-3p*, *miR-294-3p*, and *miR-295-3p* after overexpression of *pri-miR-290*. (**D**) RT-qPCR analysis of pluripotency-associated genes and three germ layer-associated gene expression in three cell lines. (**E**) Western blot analysis for phosphorylated and total forms of p38 MAPK and ERK in rPSCs, rPSCs(-SB), and the pri-miR-290-pcDNA3.1-EGFP group. (**F**) Quantification of p38 MAPK, p-p38 MAPK, ERK, and p-ERK protein intensity analysis in rPSCs, rPSCs(-SB), and the pri-miR-290-pcDNA3.1-EGFP group. (**G**) RT-qPCR analysis of up-/down-regulating target gene expression of the p38 MAPK signaling pathway in rPSCs(-SB) and the pri-miR-290-pcDNA3.1-EGFP group. (**H**) Morphology of EB induction by the pri-miR-290-pcDNA3.1-EGFP group in Day 2, Day 4, and Day 6. Scale bars: 100 µm. (**I**) Statistics of the diameter of EB formed by rPSCs and the pri-miR-290-pcDNA3.1-EGFP group. (**J**) RT-qPCR analysis of endoderm-, mesoderm-, and ectoderm-associated gene expression in rPSCs-EB-D3 and pri-miR-290-pcDNA3.1-EGFP-EB-D3. (**K**) Mature teratoma from rPSCs. Left: endoderm, gland-like cells. Middle: mesoderm, cartilage-like cells. Right: ectoderm, neural-like cells. The sections were stained with H&E. Scale bars: 100 µm. The above experiments included three replications. Error bars are SEM. Significance was tested with two-tailed Student’s *t*-tests, with * *p* < 0.05, ** *p* < 0.01, *** *p* < 0.001, and ns at *p* > 0.05.

Interestingly, all members of the miR-290 family are spliced from *D7Ertd143e* (2232 nucleotides, Genbank accession number: NR_028425.1), which is the primary molecule of this family [54]. So, in order to further explore the role of the miR-290 family in maintaining stem cell pluripotency, we cloned the *pri-miR-290* cDNA (D7Ertd143e) sequence and successfully constructed the overexpression vector pri-miR-290-pcDNA3.1-EGFP (Figure S6A), which was verified by sequencing analysis (Figure S6B). The rPSCs(-SB) were transfected with pri-miR-290-pcDNA3.1-EGFP by liposome transfection and selected with G418 (Figure 7A). Notably, rPSCs(-SB) displayed domed morphology and could be passaged over 15 after G418 selection (Figure 7A). RT-qPCR assays showed that the mRNA level of *pri-miR-290* in the pri-miR-290-pcDNA3.1-EGFP group was nearly 2-fold higher than that in the control vector group (Figure 7B). Furthermore, the expression of mature miR-290 family members, including *miR-290a-3p*, *miR-291a-3p*, *miR-291b-3p*, *miR-292a-3p*, *miR-294-3p*, and *miR-295-3p*, was significantly higher in the pri-miR-290-pcDNA3.1-EGFP group (Figure 7C).

To investigate whether the miR-290 family can replace SB203580 to maintain pluripotency in stem cells, we examined the expression levels of pluripotency markers and three germ layer markers. RT-qPCR analysis showed that the expression of pluripotency markers *Oct4* and *Sox2* in the pri-miR-290-pcDNA3.1-EGFP group was comparable to that in rPSCs, with higher expression compared with rPSCs(-SB). Furthermore, the mesoderm markers *T*, *Hand*, and *Evx1* were significantly increased in the pri-miR-290-pcDNA3.1-EGFP group (Figure 7D). In contrast, the expression of endoderm markers *Gata6* and *Sox17* was much lower in the pri-miR-290-pcDNA3.1-EGFP group. However, the pri-miR-290-pcDNA3.1-EGFP group displayed intermediate levels of ectoderm markers, such as *Nestin*, *Ncam*, and *K8*, between those observed in rPSCs and rPSCs(-SB) (Figure 7D). To further demonstrate the involvement of miR-290 in repressing the p38 MAPK signaling pathway, we assessed the protein expression of both phosphorylated and total forms of p38 MAPK and ERK. Western blot analysis revealed that p38 MAPK signaling was increased in rPSCs(-SB) compared with the rPSCs and pri-miR-290-pcDNA3.1-EGFP group, particularly evident for phosphorylated forms of the p38 MAPK protein (Figure 7E,F). However, there were no significant differences in the expression of ERK and p-ERK in rPSCs, rPSCs(-SB), and the pri-miR-290-pcDNA3.1-EGFP group (Figure 7E,F). Furthermore, we found that the down-regulating target genes of the p38 MAPK signaling pathway, *Atf3*, *Fzd*4, and *Hey1,* were significantly upregulated in the pri-miR-290-pcDNA3.1-EGFP group. Conversely, up-regulating target genes *Atm*, *Mertk*, *Sox13*, and *Stag2* were significantly downregulated compared with those expressed in rPSCs(-SB) (Figure 7G), indicating the p38 MAPK signaling pathway was inhibited.

In addition, to assess the differentiated potency of the pri-miR-290-pcDNA3.1-EGFP group in vitro, we compared the formation of EB between rPSCs and the pri-miR-290-pcDNA3.1-EGFP group. The EB derived from the pri-miR-290-pcDNA3.1-EGFP group presented a smaller diameter compared with the one derived from rPSCs (Figure 7H), with the diameter of pri-miR-290-pcDNA3.1-EGFP-EB at day 6 measuring approximately 180 μm, which was smaller than that of rPSCs-EB (Figure 7I). RT-qPCR results showed that expression levels of germ layer markers were all increased in the pri-miR-290-pcDNA3.1-EGFP group-derived EB (pri-miR-290-pcDNA3.1-EGFP-EB) compared with rPSCs-EB (Figure 7J). The formation of teratoma was also performed, demonstrating that the pri-miR-290-pcDNA3.1-EGFP group could differentiate into multiple tissues in vivo (Figure 7K).

Collectively, these results indicate that overexpression of the miR-290 family can reverse the cell phenotype induced by SB203580 deletion. Notably, the miR-290 family inhibits the p38 MAPK signaling pathway in response to SB203580 removal while leaving the ERK signaling pathway unaffected. This further supports the involvement of miR-290 in maintaining pluripotency in rPSCs through the regulation of the p38 MAPK pathway. Moreover, our results are consistent with previous studies [55,56], highlighting how the miR-290 family promotes PSC pluripotency maintenance.

## 3. Discussion

Pluripotent stem cells can be derived from different stages of early embryonic development and maintain a self-renewal state in vitro by supplementing exogenous cues [57]. EpiSCs and ESCs can be interconverted, with the transition from ESCs to EpiSCs achieved by adapting culture conditions [10,58]. However, EpiSCs reversion to ESCs is extremely inefficient, suggesting that there could be an unknown transcriptional or epigenetic barrier that prevents reversion of developmental commitments. While the differentiation of ESCs to the primed state has been achieved, the reprogramming of EpiSCs to the naïve state has mostly relied on the overexpression of transcription factors [59,60,61,62]. Recently, Yu et al. demonstrated an induced primed to naïve transition system without any exogenous transcription factor expression. They induced EpiSCs with BMP4, DOT1L inhibitor, and EZH2 inhibitor, followed by transfer into 2i/LIF culture, resulting in the conversion of primed cells into naïve colonies [26]. However, the above study requires a two-step process to complete the transition from a primed to a naïve state. In this study, we describe the derivation of a novel cell type called rPSCs from mouse EpiSCs by culturing them in rPSCs medium containing LIF, BMP4, CHIR99021, XAV939, and SB203580. We also explored the underlying mechanisms of pluripotency maintenance in rPSCs.

The rPSCs described in this study exhibit domed colonies and higher expression of pluripotency genes, as well as lower expression of the primed marker *Fgf5* compared with EpiSCs. rPSCs have the potential to form teratomas with three germ layers differentiated in vivo and can contribute to the ICM when injected into an 8-cell stage embryo. Transcriptome analysis suggests that rPSCs appear to be in an intermediate state between naïve ESCs and primed EpiSCs with unique molecular features. Collectively, we propose that the culture conditions for rPSCs promote the conversion from a prime to a naïve state, placing rPSCs in an intermediate state close to the formative state but with unique molecular features in a newly chemically defined medium.

Here, the MAPK signaling pathway was essential for maintaining pluripotency and self-renewal in rPSCs. The MAPK pathways include the ERK pathway, the Jun N-terminal kinase (JNK) pathway, and the p38 pathway. Each member of MAPK is activated in response to various extracellular stimuli, and regulating gene expression mainly controls various biological processes such as cell proliferation, cell differentiation, pluripotency, cell cycle arrest, apoptosis, etc. [63]. Several MAPK inhibitors have been investigated for their roles in early embryonic and stem cell development [64,65]. For example, the MAP2K1 inhibitor has been used to derive mouse ESCs from early blastomeres [64]. In addition, the p38 inhibitor has been reported to inhibit trophectoderm cell development in mouse morula [65]. Blocking p38α has also been found to support the naïve pluripotency of mouse ESCs [27]. In previous studies, JNK inhibitor SP600125 and p38 inhibitor SB203580 have been used to generate naïve state iPSCs from rhesus monkey fibroblasts and porcine bone marrow mesenchymal stem cells with high efficiency [66,67]. Similarly, we demonstrate that the p38 inhibitor SB203580 maintains the self-renewal of rPSCs. Withdrawal of SB203580 leads to a significant reduction in the expression of pluripotency genes, a change in cell morphology from domed to flat, and weakly positive AP staining. The addition of SB203580 during the formation of early primitive ectoderm-like cells has been shown to induce colonies exhibiting the characteristics of mouse ESC colonies with a rounded appearance and refractive edges [68].

Furthermore, the expression of endoderm-related genes, such as *Gata4*, *Gata6*, and *Sox17,* was increased after SB203580 was withdrawn. Thus, we hypothesized that activation of the p38 MAPK signaling pathway promotes differentiation towards the endoderm. Yap et al. found that p38 MAPK activity plays an important role in gastrulation and endoderm population formation; the expression of endoderm markers *Sox17*, *Ttr*, *Gata4*, *Trh,* and *Eya2* was significantly decreased in conditions with the addition of SB203580 [69]. Therefore, activation or repression of the p38 MAPK pathway at different time points of cell differentiation is critical for lineage differentiation [70]. Trouillas et al. showed that inhibition of p38 MAPK enhances neurogenesis [71], and our results showed that the withdrawal of SB203580 reduces the expression of ectoderm-related genes *Nestin*, *Pax6*, and *Ncam*. Altogether, the p38 MAPK signaling pathway plays multiple roles in PSCs and development, and the absence of SB203580 in rPSCs facilitates exit from the pluripotent state and promotes endoderm differentiation. Nevertheless, the specific mechanism of the p38 MAPK signaling pathway in regulating pluripotency and self-renewal in rPSCs requires further investigation.

Recent studies have highlighted the importance of epigenetics in pluripotency regulation. Habibi et al. found that naïve ESCs exhibit epigenetic characteristics similar to ICM, including global DNA hypo-methylation [50]. Conversely, EpiSCs exhibit a transcriptional profile and genome-wide DNA hypermethylation similar to the post-implantation epiblast [72]. The DNA methylation level in the promoter region of pluripotency genes in rPSCs was between EpiSCs and ESCs, and the mRNA levels of DNA methyltransferases (DNMT), such as *Dnmt3a*, *Dnmt3b*, and *Dnmt1,* were also significantly decreased in rPSCs. In addition, the expression of the miR-290 family was increased in rPSCs compared with EpiSCs. The MiR-290 family constitutes over 60% of the entire miRNA population in mouse ESCs [73]. Several studies have addressed the miR-290-295 cluster as a direct target of the *Oct4*, *Sox2*, and *Nanog* regulatory networks participating in stem cell regulation [73]. Our previous study demonstrated that the miR-290 family maintains developmental potential by targeting *P21* in mouse pre-implantation embryos and that knockdown of the miR-290 family in ESCs results in a significantly increased the expression of differentiation-related genes [74]. The expression of the miR-290 family is correlated with developmental potency, with a decrease in miR-290-295 expression as ESCs differentiate [45]. Jouneau et al. proposed that during the conversion of mouse ESCs from naïve to primed conditions, the expression of miR-290-295 decreased [75]. This is also supported by our results: the expression of miR-290 family members was upregulated during the conversion of primed EpiSCs to rPSCs. A super enhancer (SE) is a cluster of enhancers that has a stronger ability to promote transcription compared to the typical enhancer, and it plays an important role in the development of ESCs and multiple cancers [76]. Stelzer et al. reported that the miR-290 SE region is hypo-methylated in ESCs but becomes de novo methylated upon differentiation [47]. However, we observed higher expression levels of the miR-290 family members and methylation of the miR-290 SE region in rPSCs compared with ESCs; its function and regulation on pluripotency remain to be clarified.

Notably, the expression of the miR-290-295 cluster decreases when SB203580 is withdrawn, which is consistent with previous reports showing a slight decrease in miR-290 family miRNAs during the transition from naïve to primed state [45,77]. In recent years, the miR-290-295 cluster has been implicated in the regulation of pluripotency maintenance, self-renewal, and reprogramming of somatic cells to an ESC-like state [78]. Forced expression of *miR-291-3p*, *miR-294,* and *miR-295*, or pan-inhibition of *let7* miRNAs, improves the reprogramming efficiency of MEFs into induced pluripotent stem cells (iPSCs) by *Oct4*, *Klf4*, and *Sox2* [78,79]. Despite the well-established role of miRNAs in ESCs, their link to signaling pathways remains largely elusive. In this study, miR-290 family target genes were enriched in the MAPK signaling pathway, and *Map2k6* was identified as a common target of the miR-290 family. Therefore, we propose that the miR-290 family regulates the MAPK signaling pathway by reducing the expression of its downstream target gene *Map2k6* to maintain pluripotency when SB203580 is withdrawn. Remarkably, overexpression of *pri-miR-290* in rPSCs(-SB) changes cell morphology from flat to domed, resulting in an increase in pluripotency gene expression, a decrease in endoderm-related gene expression, and a significant upregulation of mesoderm-related genes. In human embryonic stem cells (hESCs), overexpression of miR-373 (homologous to miR-290 in mice) leads to their differentiation towards the mesendodermal lineage [80]. These results suggest that the miR-290 family can replace the p38 MAPK signaling pathway inhibitor SB203580 to maintain the pluripotency of rPSCs.

In summary, a new type of pluripotent stem cell, rPSCs, has been derived from EpiSCs in the rPSCs culture condition. The rPSCs have unique molecular features and are in intermediate states. Moreover, the miR-290 family can replace SB203580, involved in the pluripotency maintenance and self-renewal of rPSCs, by inhibiting the p38 MAPK signaling pathway. Thus, capturing unique pluripotent states in culture as expandable cell lines provides a valuable tool to understand the molecular mechanisms underpinning pluripotency transitions, germline or somatic fate determination, and cellular reprogramming, as well as the possibility of reproducing germline developmental processes in vitro [81,82]. Furthermore, understanding the interaction between the miR-290 family and the MAPK pathway opens new avenues to explore the underlying mechanisms.

## 4. Materials and Methods

### 4.1. Animals

CD-1^®^(ICR) IGS and BALB/c-nu mice were purchased from SPF (Beijing) Biotechnology (China) at the age of 6–8 weeks. All mice were housed under controlled lighting conditions (light: 08:00–20:00) and had free access to food and water.

### 4.2. Derivation of EpiSCs

Mouse gastrulas were collected from E6.5 pregnant female ICR mice. The epiblasts (E6.5) were isolated from gastrulas using a glass needle and cultured in AF medium. After 5–10 days, the outgrowths were minced into several smaller pieces using a glass needle and transferred to fresh AF medium. The colonies, named EpiSCs, were able to propagate stably with Accutase (Gibco, Gaithersburg, MD,USA) every 2 days at a ratio of 1:3–1:6, with fresh AF medium provided daily. Mouse EpiSCs were cultured in serum-free AF medium under 5% CO_2_ at 37 °C. The AF medium consisted of Activin A (20 ng/mL, R&D Systems, Minneapolis, MN, USA) and bFGF (12 ng/mL, R&D Systems) added to a basic N2B27 medium. A total of 500 mL of N2B27 medium was prepared using 240 mL DMEM/F12 (Gibco, USA), 240 mL Neurobasal (Gibco, USA), 2.5 mL N2 supplement (Gibco, USA), 5 mL B27 supplement (Gibco, USA), 1% GlutaMAX (Gibco, USA), 1% nonessential amino acids (Gibco, USA), 0.1 mM β-mercaptoethanol (Gibco, USA), 1% penicillin-streptomycin (Gibco, USA), and 5 mg/mL BSA (Gibco, USA). All culture dishes were coated with Hu Plasma Fibronectin (FN, 1 mg/mL in Dulbecco’s phosphate-buffered saline (DPBS), Millipore Billerica, MA, USA) for at least 30 min before use.

### 4.3. Conversion of EpiSCs to rPSCs

To convert EpiSCs to rPSCs, EpiSCs were dissociated into single cells using Accutase (Gibco, USA) and plated in rPSCs medium. The rPSCs medium was prepared by adding small molecules and cytokines to the N2B27 medium in the following final concentrations: 1000 U/mL LIF (Millipore, Billerica, MA, USA), 50 ng/mL BMP4 (R&D Systems, USA), 3 µM CHIR99021 (R&D Systems, USA), 10 μM SB203580 (Tocris, Minneapolis, MN, USA), and 5 μM XAV939 (Sigma, Darmstadt, Germany). After 5–7 days, domed clonal clusters emerged, which were minced into several smaller pieces using a glass needle and transferred to fresh rPSCs medium. The colonies, named rPSCs, could be stably propagated by Accutase (Gibco, USA) daily at a ratio of 1:3–1:6. All culture dishes were coated with FN for at least 30 min before use.

### 4.4. Culture of Mouse ESCs

Mouse ESCs were maintained under 5% CO_2_ at 37 °C on FN-coated dishes in 2i/LIF medium that contained serum-free N2B27 medium supplemented with 1000 U/mL LIF (Millipore, USA), 3 µM CHIR99021 (R&D Systems, USA), and 1 μM PD0325901 (R&D Systems, USA). Cells were passaged every 2 days using Accutase (Gibco, USA).

### 4.5. AP Staining

Prior to staining, cells were placed in a four-well plate, washed with DPBS, and then fixed in 4% paraformaldehyde at room temperature for 30 min. The cells were washed with DPBS again, followed by the addition of an AP staining solution. The AP staining solution was prepared as follows: 50 µL sodium nitrite solution was gently mixed with 50 µL FRV-alkaline solution and incubated at 37 °C for 3 min; then, 2.25 mL H_2_O and 50 µL naphthol-As-BI alkaline solution were added to the mixture. The fixed cells were incubated overnight in the dark with the staining solution.

### 4.6. Karyotype Analysis

The cell cultures were prepared to achieve a confluence of 70–80% on the day of sampling. After 2 h of incubation in fresh medium, a Colcemid solution was added to the medium at a final concentration of 0.02 mg/mL and incubated for 2 h. Then, the cells were washed with DPBS, and Accutase was used to dissociate the cells. The suspensions were centrifuged at 1500 rpm for 5 min to collect the tested cells. The cell pellets were gently resuspended in an 8 mL hypotonic solution of 0.075 mol/L KCl (Sigma, USA) and incubated at 37 °C in a water bath for 40 min. Subsequently, 1 mL of ice-cold fixative liquid (methanol: glacial acetic acid = 3:1) was added to the resuspended cells and mixed gently, and the solution was then centrifuged at 1000 rpm for 10 min. After discarding the supernatant, the cells were gently mixed in an 8 mL fixative solution and incubated in a 37 °C water bath for 30 min to fix the cells. This process was repeated twice. Finally, the pellet was re-suspended in a final volume of 1 mL of fixative and dropped onto ice-cold glass slides, which were then dried for 1 h at 70 °C in a drying oven. The glass slides were stained in Giemsa (Sigma, USA) for 10 min, washed with distilled water, and air-dried. For each analysis, at least 60–80 metaphases were examined to determine the number of chromosomes and the presence of structural chromosomal abnormalities.

### 4.7. Immunofluorescence

The cells used for immunofluorescence assays were washed with DPBS, fixed in 4% paraformaldehyde for 30 min at room temperature, and permeabilized with 0.1% Triton X-100 (Sigma, USA) and 1% BSA in DPBS for 30 min. The cells were then incubated with primary antibodies overnight at 4 °C. After the cells were washed three times in 1% BSA and 0.1% Triton X-100 in DPBS for 5 min per wash, they were incubated with secondary antibodies for 1 h at room temperature in the dark. The cells were washed three times in 1% BSA and 0.1% Triton X-100 in DPBS for 5 min per wash. Nuclei were stained with 10 μg/mL DAPI, and then cells were mounted on glass slides with Vectashield. The antibodies used are listed in Supplementary Table S1.

### 4.8. Total RNA, miRNA Extraction, and RT-qPCR

Total RNA and miRNA were extracted using an RNeasy Mini Kit (No. 74104, Qiagen, Hilden, Germany) and an miRNeasy Micro Kit (No. 217084, Qiagen, Germany), respectively. Reverse transcription was performed using a PrimeScript RT reagent kit with gDNA Eraser (RR047A, Takara, Otsu, Shiga, Japan) and a Mir-X miRNA First-Strand Synthesis kit (638313, Clontech, Mountain View, CA, USA). Real-time quantitative polymerase chain reaction (RT-qPCR) was performed using GoTaq^®^ qPCR Master Mix (A6002, Promega, Madison, WI, USA), and signals were detected with an ABI7500 Real-Time PCR System (Life Technologies, Singapore). All experiments were performed according to the respective manufacturer’s protocols. Relative gene expression was determined using the 2^−ΔΔCt^ method. The primers used in these analyses are listed in supplementary Table S2. They were designed using Primer Premier 5.0 and synthesized by the Beijing Genomics Institution (BGI, Beijing, China).

### 4.9. Western Blot

Whole-cell protein extracts were isolated from cells using RIPA lysis buffer (P0013B, Beyotime Technology, Shanghai, China) supplemented with protease inhibitor cocktail (78443, Thermo Fisher Scientific, Waltham, MA,) and phosphatase inhibitor cocktail. The cell lysates were placed on ice for 10 min and centrifuged at 12,000 rpm at 4 °C for 10 min, and the supernatant was transferred to new tubes. Protein concentration was determined using a Pierce BCA Protein Assay Kit (23225, Thermo, Waltham, MA, USA,). Subsequently, 20 μg of protein was separated via SDS polyacrylamide gel electrophoresis (SDS-PAGE). Blots were incubated in 5% BSA/TBST at room temperature for 2 h, and then they were incubated with the stated antibodies in 2% skimmed milk powder/TBST at 4 °C overnight. The membranes were washed three times in TBST solution and incubated with secondary antibodies (diluted in 2% milk powder in TBST solution) for 1 h at room temperature with shaking. Bound antibodies were detected with Clarity Western ECL Substrate (170-5060, Bio-Rad, Hercules, CA, USA). The intensity of the protein bands was calculated using ImageJ software (v1.50i). The antibodies used are listed in Supplementary Table S1.

### 4.10. Generation of Teratoma

Cells were disaggregated using Accutase into small cell clusters and resuspended in 100 μL DPBS. A total of 5 × 10^6^ cells were injected under the epithelium of BALB/c-nu mice. The tumors were allowed to develop for 4 weeks, then fixed in 4% paraformaldehyde and processed for paraffin sectioning. Sections were observed following hematoxylin and eosin staining.

### 4.11. Formation of an Embryonic Body

Cells on the FN-coated plate were incubated with Accutase for 3 min at 37 °C until the colonies were completely disaggregated. The colonies were then resuspended in EB medium, containing DMEM/F12 + 20% Knockout serum replacement (KSR, Gibco, Gaithersburg, MD, USA) + 1% GlutaMax + 1% NEAA + 1% β-mercaptoethanol. The cells were resuspended in embryoid body medium at 1 × 10^5^/mL and cultured in suspension at 30 μL per drop for 6 days.

### 4.12. Generation of Chimera

To generate chimeric embryos, donor cells were microinjected into the 8-cell ICR mice using a piezo-assisted micromanipulator attached to an inverted microscope. They developed into blastocysts after being grown in potassium simplex optimization medium (KSOM, Millipore, Billerica, MA, USA). Recover the injected embryos in KSOM culture for 24 h to form blastocysts.

### 4.13. DNA Methylation Analysis

Total DNA was extracted using the Wizard^®^ SV Genomic DNA Purification System (A2360, Promega, Madison, WI, USA). To determine the DNA methylation profiles of the *Oct4*, *Sox2*, *Nanog* promoter regions, and the pri-miR-290 super enhancer region, sample pools consisting of genomic DNA from cells were subjected to the bisulfite reaction using the EZ DNA Methylation-GoldTM Kit (D5005, ZYMO RESEARCH, Irvine, CA, USA), according to the manufacturer’s instructions. Each bisulfite-treated genome was amplified using GoTaq^®^ Hot Start Master Mix (M5122, Promega, Madison, WI, USA) and the specific primers listed in Supplementary Table S2. Bisulfite-treated genomic mixtures were subjected to PCR, and 20 of the resulting subclones were sequenced with bisulfite for each sample.

### 4.14. Transcriptome Analysis

All collected cells were treated with the RNeasy Mini Kit (No. 74104, Qiagen, Hilden, Germany) to obtain mRNA. RNA integrity was assessed using the RNA Nano 6000 Assay Kit of the Agilent Bioanalyzer 2100 system (Agilent Technologies, Palo Alto, CA, USA). Sequencing libraries were generated using the Hieff NGS Ultima Dual-mode mRNA Library Prep Kit for Illumina (Yeasen Biotechnology (Shanghai), China), and index codes were added to attribute sequences in each sample. The cDNA libraries were sequenced using the Illumina NovaSeq platform. Genes with an adjusted *p*-value < 0.01 and a fold change of ≥2 found by DESeq2 were assigned as differentially expressed. Principal component analysis (PCA), GO enrichment analysis, Pearson’s correlation analysis, and pathway enrichment analysis of differential genes were performed using an online data analysis platform (https://www.biocloud.net/ accessed on 10 May 2022). The volcano plot was performed using the ggplot2 R package (v3.3.3), and heatmaps of selected genes were plotted using the pheatmap R package (v1.0.12).

### 4.15. Construction of Expression Plasmid Vectors

The pri-miR-290 sequence was PCR-amplified from mouse ESCs cDNA using LA Taq (RR02MA, Takara, Otsu, Shiga, Japan). The pri-miR-290 cDNA was cloned into Nhe I/Kpn I (R6501 and R6341, Promega, Madison, WI, USA) restriction enzyme sites in a pcDNA3.1-EGFP plasmid (No. 129020, Addgene, Cambridge, MA, USA). Please refer to our previous published papers for details [74]. The primers are listed in Supplementary Table S2.

### 4.16. Dual-Iuciferase Reporter Assay

ESCs were co-transfected with pmirGLO plasmids containing wild or mutant 3′-untranslated region (3′-UTR) fragments from *Map2k6* (or *Map2k3*) and miRNA mimics using Lipofectamine 2000 (Invitrogen, Carlsbad, CA, USA), following the manufacturer’s protocol. After 24 h of transfection, firefly and renilla luciferase activities were measured consecutively using a GloMax^®^ 20/20 Luminometer (Promega, Madison, WI, USA). The ratio of firefly to renilla luciferase luminescence was calculated. The sequences are listed in Supplementary Table S2.

### 4.17. miR-290 Family Target Genes Analysis

The GO and KEGG analyses of the miR-290 family target genes were performed using DAVID software available at https://david.ncifcrf.gov/summary.jsp (accessed on 28 December 2020), following the website operation process.

### 4.18. Statistical Analysis

All measures were taken from distinct samples. Unless there was a specific statement about the number of replicates, three replicates were analyzed for each experiment. All data were reported as the mean ± s.e.m. The Student’s *t*-test was used for comparisons between two groups, with * *p* < 0.05, ** *p* < 0.01, *** *p* < 0.001, and ns at *p* > 0.05.

## Data Availability

Our rPSCs RNA-seq data are available through the NCBI Sequence Read Archive under the ID PRJNA989402. The RNA-seq data for ESCs and EpiSCs were obtained from NCBI (accession number: GSE99494, accessed on 6 May 2023). FSCs were obtained from NCBI (accession number: GSE131556, accessed on 6 May 2023). Moreover, 2-Cell, 4-Cell, and E3.5 ICM were obtained from NCBI (accession numbers: GSE126056 and GSE66582, accessed on 6 May 2023). E4.0 ICM, E5.5 EPI, and E6.5 EPI were obtained from NCBI (accession number: GSE76505, accessed on 6 May 2023).

## Supplementary Materials

The following supporting information can be downloaded at: https://www.mdpi.com/article/10.3390/ijms25052681/s1.
