# Supplementary material for: MiR-290 Family Maintains Pluripotency and Self-Renewal by Regulating MAPK Signaling Pathway in Intermediate Pluripotent Stem Cells
Source: Int J Mol Sci. 2024 Feb 26;25(5):2681. doi: 10.3390/ijms25052681 (PMC10931995; doi:10.3390/ijms25052681)
Supplement: Supplementary file 1 [file ijms-25-02681-s001.zip › ijms-2723403-supplementary.pdf]

Supplementary Materials

This supplementary materials file contains the following contents:

Contents	Details
Supplementary Figures	Figure S1, S2, S3, S4, S5 and S6
Supplementary Tables	Table S1 and S2

Figure S1

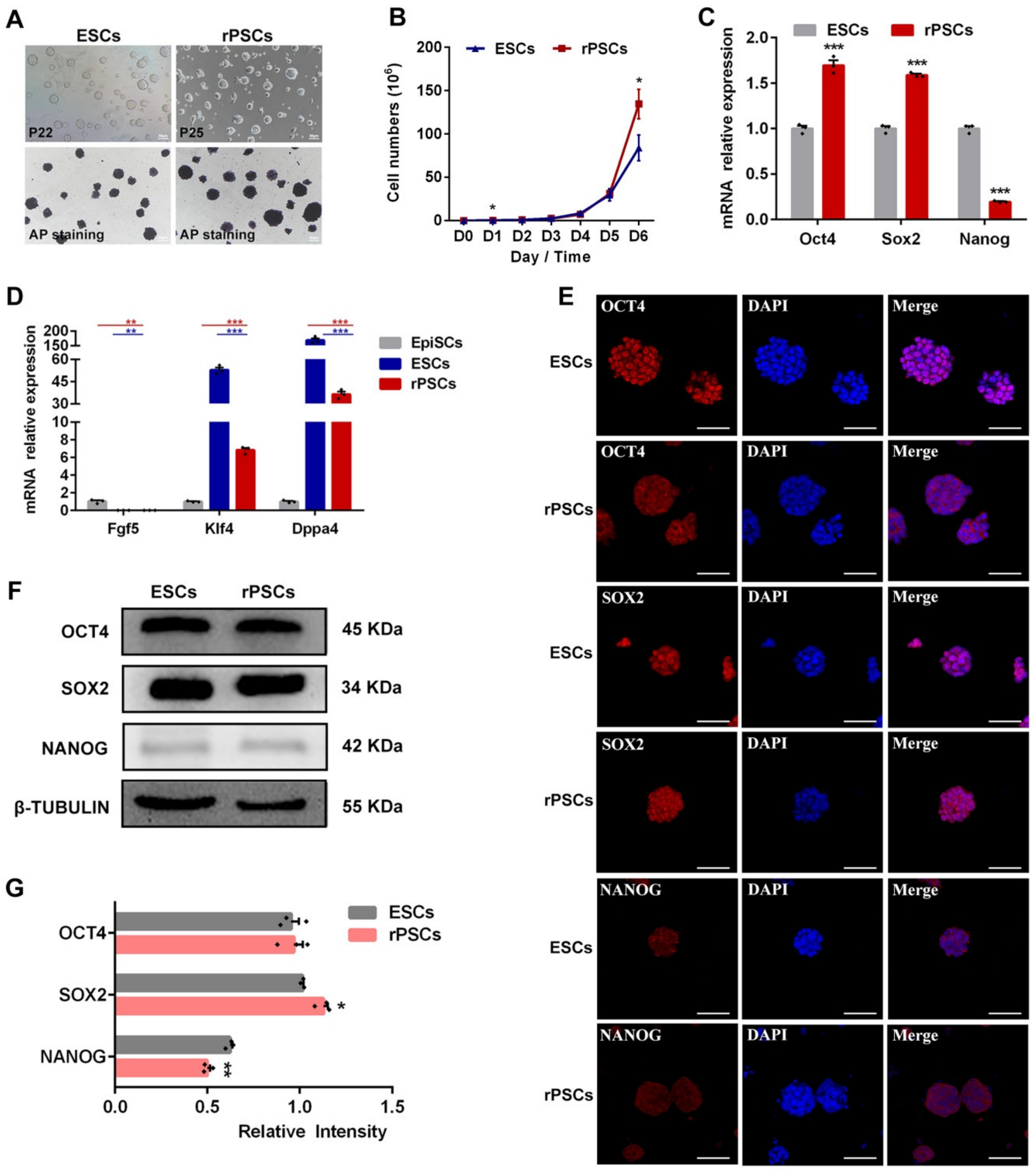

Figure S1 Characterization of rPSCs.

(A) AP staining on rPSCs and ESCs. Scale bars: 100 μm. **(B)** Cell growth curve of rPSCs and ESCs. **(C)** Relative expression of *Oct4*, *Sox2*, and *Nanog* measured by RT-qPCR in rPSCs and ESCs. **(D)** Relative expression of *Fgf5*, *Klf4*, and *Dppa4* measured by RT-qPCR in rPSCs, ESCs, and EpiSCs. **(E)** Immunofluorescence staining of OCT4, SOX2, and NANOG in rPSCs and ESCs. Scale bars: 50 μm. **(F)** Western blot analysis for OCT4, SOX2, and NANOG in rPSCs and ESCs. **(G)** Quantification of OCT4, SOX2, and NANOG protein intensity analysis in rPSCs and ESCs. The above experiments included three replicated. Error bars are SEM. Significance was tested with a two-tailed Student's *t*-tests, with $*p < 0.05$, $**p < 0.01$, $***p < 0.001$.

Figure S2

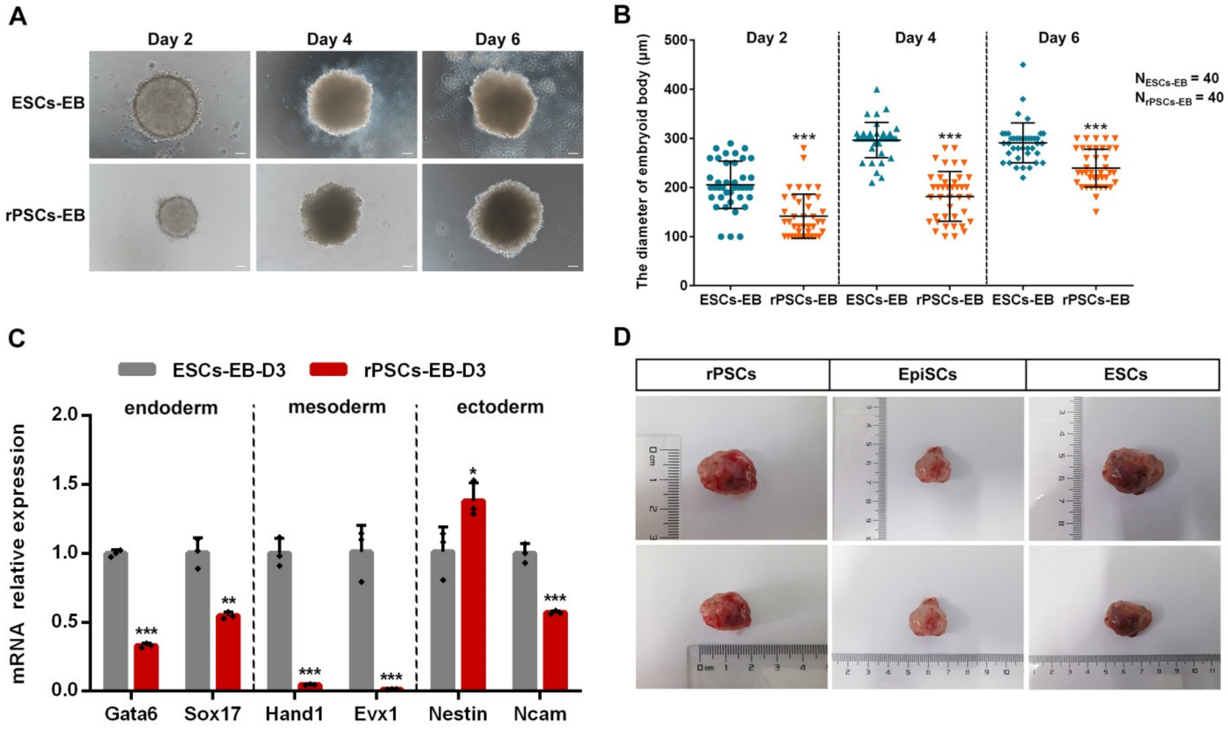

Figure S2 Differentiation potential of rPSCs.

(A) Morphology of embryoid body induction by rPSCs and ESCs in Day 2, Day 4, and Day 6. Scale bars: 100 μm. **(B)** Statistics of the number and diameter of embryoid body formed by rPSCs and ESCs. N: represents the number of EB spheres. **(C)** RT-qPCR analysis of endoderm, mesoderm and ectoderm associated genes expression in the embryoid body of rPSCs and ESCs. **(D)** Teratoma morphology of rPSCs, ESCs, and EpiSCs. The above experiments included three replicated. Error bars are SEM. Significance was tested with a two-tailed Student's *t*-tests, with $*p < 0.05$, $**p < 0.01$, $***p < 0.001$.

Figure S3

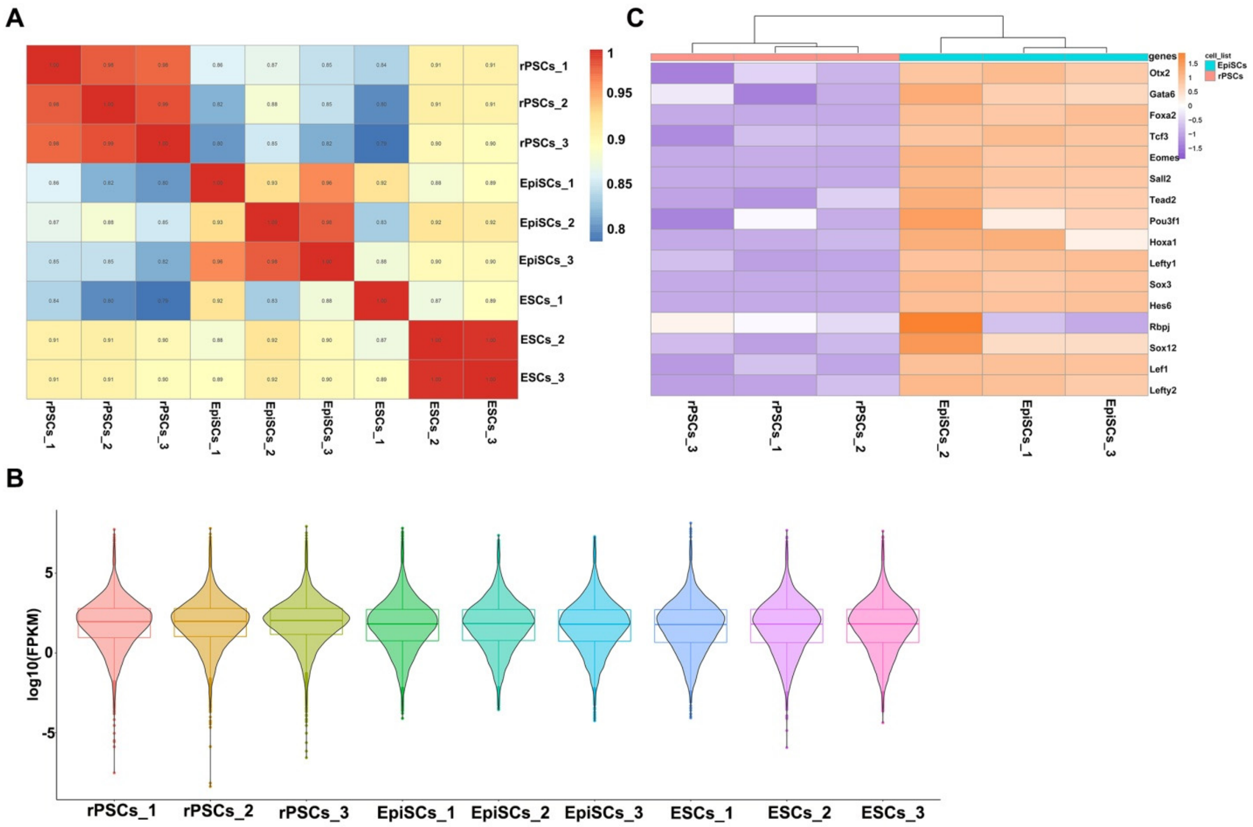

Figure S3 Molecular features of rPSCs.

(A) The correlation matrix of gene expression was clustered using Pearson correlation. **(B)** Violin plot showed the expressed genes in rPSCs, ESCs, and EpiSCs. **(C)** Heatmap showed primed pluripotent markers in rPSCs and EpiSCs based on RNA-seq data.

Figure S4

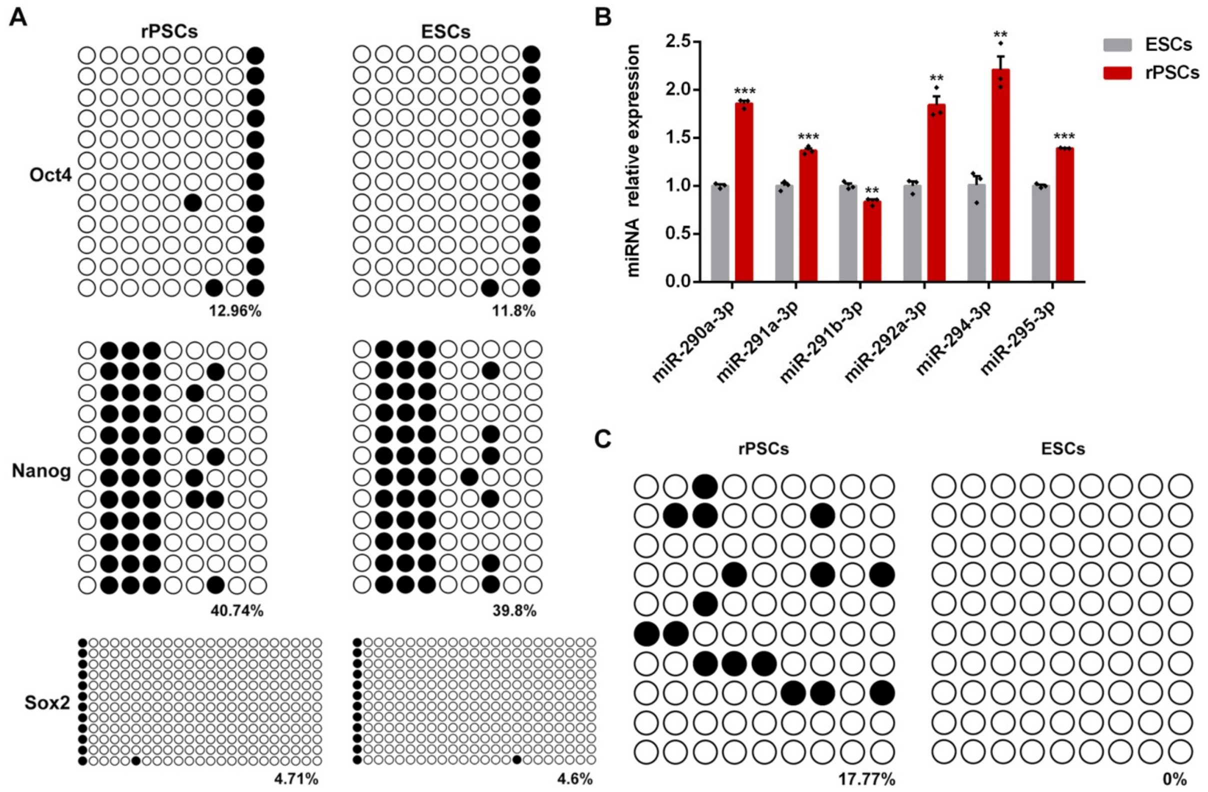

Figure S4 Epigenetics changes during EpiSCs to rPSCs transition.

(A) DNA methylation analysis of *Oct4*, *Sox2*, and *Nanog* promoter regions in rPSCs and ESCs. **(B)** RT-qPCR analysis of mature miRNAs *miR-290-3p*, *miR-291a-3p*, *miR-291b-3p*, *miR-292a-3p*, *miR-294-3p*, and *miR-295-3p* in rPSCs and ESCs. **(C)** DNA methylation analysis of miR-290 super enhancer (SE) region in rPSCs and ESCs. The above experiments included three replicated. Error bars are SEM. Significance was tested with a two-tailed Student's *t*-tests, with $^{**}p < 0.01$, $^{***}p < 0.001$.

Figure S5

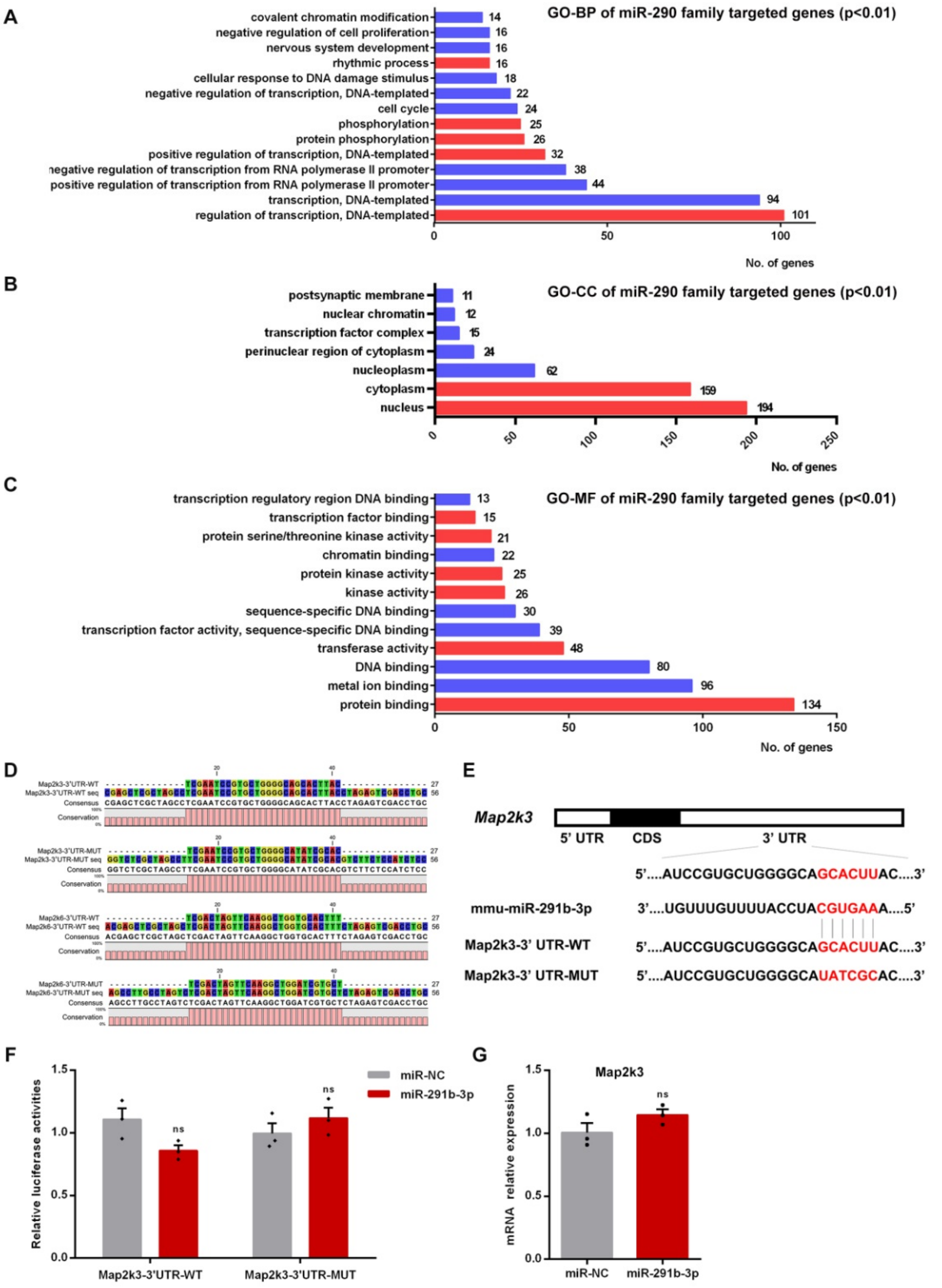

Figure S5 Screening of candidate target genes of miR-291b-3p.

(A-C) GO-BP, GO-MF, and GO-CC analyses of candidate genes. The above experiments included three replicated. **(D)** DNA sequence of Map2k3-3'UTR-WT, Map2k3-3'UTR-MUT, Map2k6-3'UTR-WT, and Map2k6-3'UTR-MUT cloned in pmiGLO vector was confirmed by sequencing. No mutation exists in sequence. **(E)** Target binding site of *miR-291b-3p* in the Map2k3 mRNA 3′-UTR. CDS, coding sequence; WT, wild-type seed sequence; MUT, mutant seed sequence. **(F)** Relative luciferase activity in ESCs co-transfected with *miR-291b-3p* mimic and Map2k3–3′UTR-WT or Map2k3–3′UTR-MUT luciferase reporter vector. Each experiment included 1 × 10^6 cells. **(G)** RT-qPCR analysis the expression levels of *Map2k3* in ESCs after *miR-291b-3p* mimic transfection. Error bars are SEM. Significance was tested with a two-tailed Student's *t*-tests, ns at $p > 0.05$.

Figure S6

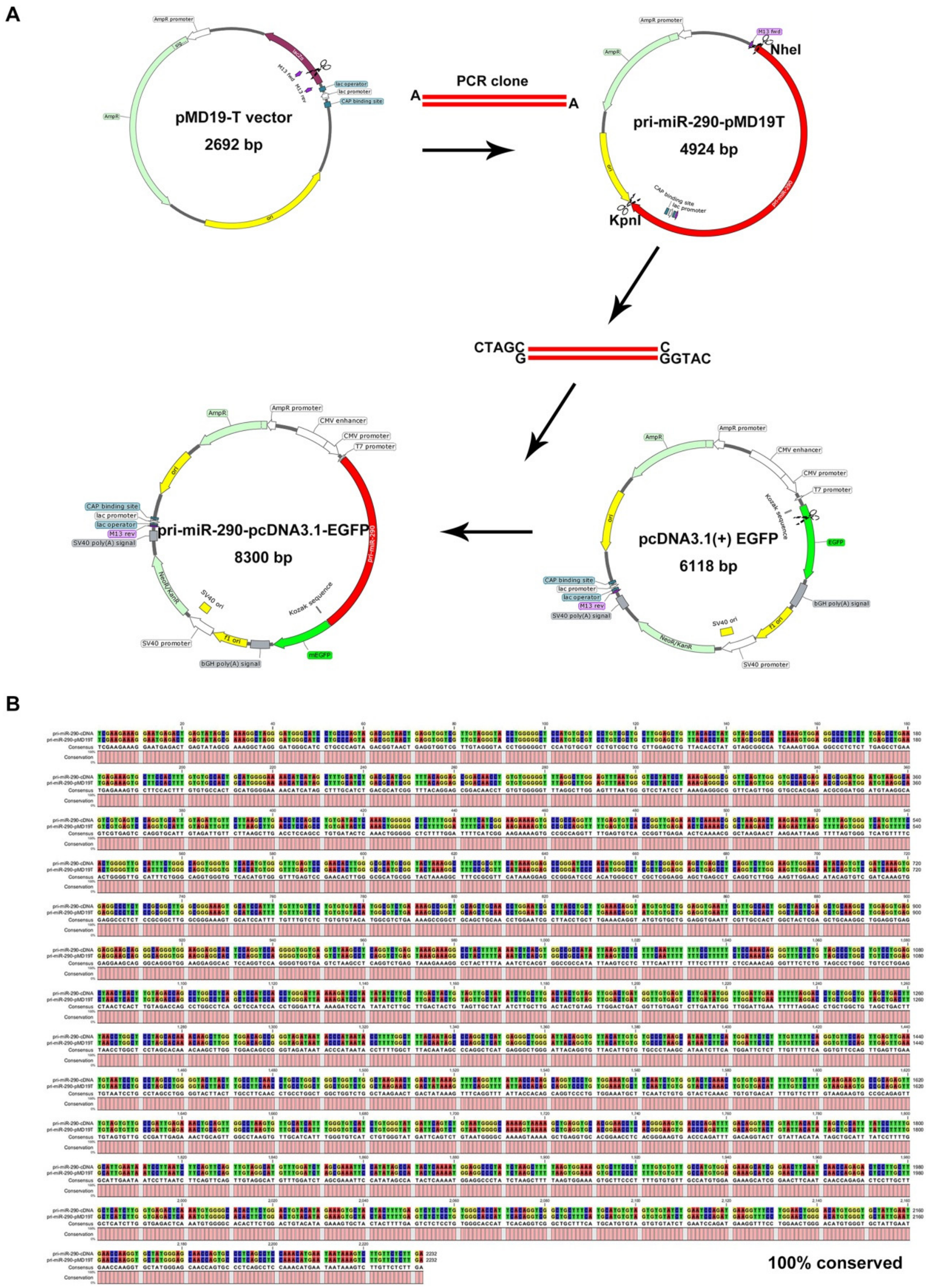

Figure S6 pri-miR-290-pcDNA3.1-EGFP overexpression vector construction.
(A) Schematic representation of pri-miR-290-pcDNA3.1-EGFP plasmid. **(B)** DNA sequence of pri-miR-290 cDNA confirmed by sequencing. No mutation existing in pri-miR-290 cDNA sequence.

Supplementary Material

Table S1 Antibody list

Antibodies	Source	Identifier
Rabbit polyclonal anti-OCT4	Abcam	Cat No: ab19857
Mouse monoclonal anti-SOX2	Abcam	Cat No: ab79351
Rabbit polyclonal anti-NANOG	Proteintech	Cat No: 14295-1-AP
Rabbit polyclonal anti-AFP	Proteintech	Cat No: 14550-1-AP
Rabbit polyclonal anti-SMA	Proteintech	Cat No: 14395-1-AP
Rabbit polyclonal anti-GFAP	Proteintech	Cat No: 16825-1-AP
Rabbit polyclonal anti-GATA6	Cell signaling	Cat No: 5851
Goat polyclonal anti-T	Snata Cruz	Cat No: sc17743
Goat polyclonal anti-NESTIN	Snata Cruz	Cat No: sc21247
Rabbit polyclonal anti-H3K27me3	Abcam	Cat No: ab192985
Rabbit polyclonal anti-H3K4me3	Abcam	Cat No: ab8580
Rabbit polyclonal anti-H3K9me3	Abcam	Cat No: ab8898
Rabbit polyclonal anti-MAP2K6	Proteintech	Cat No: 12745-1-AP
Rabbit polyclonal anti-p38 MAPK	Proteintech	Cat No: 14064-1-AP
Rabbit polyclonal anti-Phospho-p38 MAPK	Proteintech	Cat No: 28796-1-AP
Rabbit polyclonal anti-ERK1/2	Proteintech	Cat No: 11257-1-AP
Rabbit polyclonal anti-Phospho-ERK1/2	Proteintech	Cat No: 28733-1-AP
Rabbit polyclonal anti-β-TUBULIN	Abcam	Cat No: ab15568

Supplementary Material

Table S2 Primer sequences

**RT-qPCR primers**	
**Gene name**	**Sequences**
GAPDH	Forward: AAAATGGTGAAGGTCGGTGTG
	Reverse: AATGAAGGGGTCGTTGATGC
Oct4	Forward: CTGAGGGCCAGGCAGGAGCACGAG
	Reverse: CTGTAGGGAGGGCTTCGGGCACTT
Sox2	Forward: GGCAGCTACAGCATGATGCAGGAGC
	Reverse: CTGGTCATGGATTGTACTGCAGG
Nanog	Forward: CAGGTGTTTGAGGGTAGCTC
	Reverse: CGGTTCATCATGGTACAGTC
Gata6	Forward: TGCTGGAAATTGCAACAAACC
	Reverse: GTCACGTGGTACAGGCGTCA
Sox17	Forward: GTCAACGCCTTCCAAGACTTG
	Reverse: GTAAAGGTGAAAGGCGAGGTG
Gata4	Forward: CCCTACCCAGCCTACATGG
	Reverse: ACATATCGAGATTGGGGTGTCT
T	Forward: GCTTCAAGGAGCTAACTAACGAG
	Reverse: CCAGCAAGAAAGAGTACATGGC
Hand1	Forward: CTACCAGTTACATCGCCTACTTG
	Reverse: ACCACCATCCGTCTTTTTGAG
Evx1	Forward: GAGAGCCGAAAGGACATGGTT
	Reverse: CTGCCTGCTAGTCCATCGAC
Flk1	Forward: TTTGGCAAATACAACCCTTCAGA
	Reverse: GCTCCAGTATCATTTCCAACCA
Nestin	Forward: CCACAGTGCCCAGTTCTACTG
	Reverse: TAAAATAGAGTGGTGAGGGTTGA
K8	Forward: TCCATCAGGGTGACTCAGAAA

	Reverse: TTCACCGTGACGGCTGTG
Pax6	Forward: TACCAGTGTCTACCAGCCAAT
	Reverse: TGCACGAGTATGAGGAGGTCT
Ncam	Forward: GGGGAGGATGCTGTGATTGTC
	Reverse: GCGGTAAGTACCCTCATCTGT
Fgf5	Forward: AAGTAGCGCGACGTTTTCTTC
	Reverse: CTGGAAACTGCTATGTTCCGAG
Klf4	Forward: GTGCCCCGACTAACCGTTG
	Reverse: GTCGTTGAACTCCTCGGTCT
Dppa4	Forward: TCCTGCAAAGGCTAAAGCAACG
	Reverse: TCCTGGCGTCTCAGTGTCTTG
Dnmt1	Forward: AAGAATGGTGTTGTCTACCGAC
	Reverse: CATCCAGGTTGCTCCCCTTG
Dnmt3a	Forward: ATGTGGTTCGGAGATGGCAAG
	Reverse: AGATGGCTTTGCGGTACATGG
Dnmt3b	Forward: CGTTAATGGGAACTTCAGTGACC
	Reverse: CTGCGTGTAATTCAGAAGGCT
Dnmt3l	Forward: GCTCTAAGACCCTTGAAACCTTG
	Reverse: GTCGGTTCACTTTGACTTCGTA
Tet1	Forward: ACACAGTGGTGCTAATGCAG
	Reverse: AGCATGAACGGGAGAATCGG
Tet2	Forward: AGAGAAGACAATCGAGAAGTCGG
	Reverse: CCTTCCGTACTCCCAAACTCAT
Ezh1	Forward: CCAGACTGCCAGAATCGCTTT
	Reverse: CAGGTGCTTTTTGAGGCCA
Ezh2	Forward: AGTGACTTGGATTTTCCAGCAC
	Reverse: AATTCTGTTGTAAGGGCGACC
Wdr5	Forward: TTTGAAGATTTGGGACGTGAGTT
	Reverse: ATGGGCAGGCAAAGTCTTGAG

Braf	Forward: CAATTGAGCCTGTGAATATCGATGAA
	Reverse: TATTTCCGAAGACAGCGCATCAG
Chuk	Forward: AGCATTCAGCTTGACTTGGAGAGATA
	Reverse: ATGACACCAACCTCAGAATAGTGAATG
Stk3	Forward: TGGATGGCTCCTGAGGTAATTCA
	Reverse: CTGAATGTTGGTGGTGGGTTTGTA
Map3k3	Forward: TTCGGTGTCAGTCCTGTCTTACCA
	Reverse: GAACAGCATAAGAGTGACTCCAACTTG
Dusp9	Forward: AGAGTCTGAGTCGGTCATGC
	Reverse: ACTGCTACCCTGGTCGTACA
Mk3	Forward: ATGACAAGTCCTGTGATATGTGGTCC
	Reverse: ACCATTCAGGGTTAGGGAAGCTATAC
Atf3	Forward: TTGAGGATTTTGCTAACCTGACACC
	Reverse: TTGTTGACGGTAACTGACTCCAGC
Fzd4	Forward: TCGGCTACAACGTGACCAAGATG
	Reverse: CTGGAGCAGCCGTACTGGATGA
Hey1	Forward: GACGAGACCGAATCAATAACAGTTTG
	Reverse: CAGGTGATCCACAGTCATCTGCA
Atm	Forward: AACTTTGGTTTACTTGAGGCCATCA
	Reverse: AGGACTAGAAGGTTTACAGGCTGAGC
Mertk	Forward: CAGGGCCTTTACCAGGGAGACT
	Reverse: GCTGTGTGTGCTGGATGTGATCTT
Sox13	Forward: GATGCCACCAACGCTAAAGCTC
	Reverse: CTGTAAGTTGCGGTTGAAGTCCAG
Stag2	Forward: CCTACAAGCATGACCGGGACATA
	Reverse: ATGTCTGAACATCTCTGCTGTGACG
Map2k3	Forward: GCCTCAGACCAAAGGAAAATCC
	Reverse: GGTGTGGGGTTGGACACAG
Map2k6	Forward: ATGTCTCAGTCGAAAGGCAAG

	Reverse: TTGGAGTCTAAATCCCGAGGC
pri-miR-290	Forward: TCGAAGAAAGGAATGAGACTGAGTA
	Reverse: TCAAGAGAACAAGACTTTATTATTC
**miRNA RT-qPCR primers**	
**Gene name**	**Sequences**
U6	Forward: TGGAACGCTTCACGAATTTGCG
	Reverse: GGAACGATACAGAGAAGATTAGC
miR-290a-3p	Forward: AAAGTGCCGCCTAGTTTTAAGCCC
miR-291a-3p	Forward: AAAGTGCTTCCACTTTGTGTGC
miR-291b-3p	Forward: AAAGTGCATCCATTTTGTTTGT
miR-292a-3p	Forward: CAAAGTGCCGCCAGGTT
miR-294-3p	Forward: CGAAAGTGCTTCCCTTTTGT
miR-295-3p	Forward: AAAGTGCTACTACTTTTGAGTCT
**Gene clone primers**	
**Gene name**	**Sequences**
pri-miR-290 cDNA	Forward: AAAGAGCTCTCGAAGAAAGGAATGAGACTGAGTA
	Reverse: AAACCGCGGTCAAGAGAACAAGACTTTATTATTC
**DNA methylation primers**	
**Gene name**	**Sequences**
BS-Oct4	Forward: GTTTTGGATATGGGTTGAAATATTG
	Reverse: CCCCACCTAATAAAAATAAAAAAAC
BS-Sox2	Forward: GGGTTTTGTTTTATTTTGGTTTTAGTTT
	Reverse: TCTCTTCTCTACCTTAACAACTCCTAAT
BS-Nanog	Forward: TAGGATATAGGTTTTTTTTTAGATTTG
	Reverse: AAAACAAAACACCAACCAAATCAAAATA
BS-pri-miR-290 SE	Forward: AGATGGTAAGTATATGTTTGGGGG
	Reverse: TAATCTCACTATATAAACCTAACCTACC
**miRNA mimics sequences**	

Gene name	Sequences
miR-291b-3p	AAAGUGCAUCCAUUUUGUUUGU
**Luciferase reporter assays**	
**Gene name**	**Sequences**
Map2k3-3´UTR-WT	Forward: TCGAATCCGTGCTGGGGCAGCACTTAC
	Reverse: CTAGGTAAGTGCTGCCCCAGCACGGAT
Map2k3-3´UTR-MUT	Forward: TCGAATCCGTGCTGGGGCATATCGCAC
	Reverse: CTAGGTGCGATATGCCCCAGCACGGAT
Map2k6-3´UTR-WT	Forward: TCGACTAGTTCAAGGCTGGTGCACTTT
	Reverse: CTAGAAAGTGCACCAGCCTTGAACTAG
Map2k6-3´UTR-MUT	Forward: TCGACTAGTTCAAGGCTGGATCGTGCT
	Reverse: CTAGAGCACGATCCAGCCTTGAACTAG
